# Chemical and Sensory Profiles of Sauvignon Blanc Wine Following Protein Stabilization Using a Combined Ultrafiltration/Heat/Protease Treatment

**DOI:** 10.3389/fnut.2022.799809

**Published:** 2022-06-29

**Authors:** Yihe Sui, David Wollan, Jacqui M. McRae, Richard Muhlack, Dimitra L. Capone, Peter Godden, Kerry L. Wilkinson

**Affiliations:** ^1^Department of Wine Science and Waite Research Institute, The University of Adelaide, Glen Osmond, SA, Australia; ^2^The Australian Research Council Training Centre for Innovative Wine Production, Glen Osmond, SA, Australia; ^3^VAF Memstar, Nuriootpa, SA, Australia; ^4^School of Chemical Engineering and Advanced Materials, The University of Adelaide, Adelaide, SA, Australia; ^5^The Australian Wine Research Institute, Glen Osmond, SA, Australia

**Keywords:** heat stability, haze, membrane filtration, wine protein, protease, Aspergillopepsin, thaumatin-like protein, chitinases

## Abstract

Ultrafiltration (UF) was evaluated as a process by which proteins can be selectively removed from white wine as an alternative approach to protein stabilization than traditional bentonite fining. Unfined Sauvignon Blanc wine (50 L) was fractionated by UF and the retentate stabilized either by heat and/or protease treatment or bentonite fining before being recombined with the permeate. The heat stability of recombined wine was significantly improved when retentate was heated following protease (Aspergillopepsin) addition and subsequently stabilized by bentonite treatment. The combined UF/heat/protease treatment removed 59% of protein and reduced the quantity of bentonite needed to achieve protein stability by 72%, relative to bentonite treatment alone. This innovative approach to protein stabilization had no significant impact on wine quality or sensory characteristics, affording industry greater confidence in adopting this technology as a novel approach to achieving protein stability.

## Introduction

Clarity and transparency are critical outcomes of white wine production to meet consumer expectations. White wine can develop haze or precipitate during long-term storage or after heat exposure if proteins are not removed before bottling (1). Two pathogenesis-related proteins, chitinases and thaumatin-like proteins (TLPs), have been identified as the major contributors to protein haze formation (2, 3). To prevent this perceived fault, winemakers routinely use bentonite as a fining agent to remove proteins from grape juice or wine, to improve its heat stability (4). However, bentonite can also remove desirable aroma and flavor compounds, thereby having a negative effect on wine quality (2, 5, 6). The sensory and financial impacts of bentonite use have driven research into alternatives to bentonite fining (1, 3, 7–9).

A treatment combining heat and protease addition using Aspergillopepsin (AGP) was shown to be effective at removing proteins from grape juice without negative sensory impact (7). The encouraging results obtained for juice and wine, together with regulatory approval for the use of AGP during winemaking in Australia, New Zealand, the European Union and the recent approval by the International Organization of Vine and Wine (OIV), prompted further evaluation and optimization of protease heat degradation as a novel approach to protein stabilization of white wine (9, 10). Heating wine is not generally a winemaker-preferred process due to concerns about the potential negative impact on wine volatile composition and the potential for accelerating oxidation (11). Heat exposure can cause a loss of fruity and floral aromas in young white wines due to decreased volatile esters and acetates in wine (11). Additionally, storage of white wine at 40–50°C for between 7 days and 6 months (to simulate conditions experienced during shipping) was shown to promote browning and the formation of an aged aroma bouquet (11–14). However, heating juice or wine at 61°C for up to 51 min did not result in any perceivable changes to wine sensory quality (15). Thus, shorter durations of heat exposure at moderate temperatures should be less likely to negatively impact wine.

Ultrafiltration (UF) of wine with membranes that have nominal molecular weight cut-off (MWCO) specifications of 5–10 kDa can fractionate wine into heat-stable permeate and protein-enriched retentate (9). Fractionation enables targeted treatment, thereby mitigating any negative quality effects, compared to treatment of wine. Pilot-scale trials demonstrated that haze-forming proteins are effectively retained by the membrane and that heating the retentate fraction at 62°C for 10 min, with or without AGP, significantly reduced protein concentrations and improved the heat stability of recombined wine (9). However, the efficacy of this process on a larger scale, and any sensory implications of the combined UF/heat/protease treatment, are unknown.

This study therefore sought to evaluate the impact of UF/heat/protease treatment on wine volatile and organoleptic profiles, along with protein stabilization, on a larger scale using chemical and sensory analyses. Any evidence of wine oxidation arising from treatment was also specifically assessed.

## Materials and Methods

### Wine Samples and Preparation of Heat Stability Agents

Unfined 2019 Sauvignon Blanc wine (200 L) was sourced from Pernod Ricard Winemakers (Rowland Flat, SA, Australia), sterile filtered and stored at 0^°^C prior to use.

Bentonite (SIHA Active bentonite G, Begerow, Langenlonsheim, Germany) was prepared as a 5% slurry in water and added to wine or retentate at concentrations determined by preliminary fining trials (data not shown), performed using a standard industry protocol (16). Two commercial AGP protease preparations were trialed: DSM (Royal DSM, Heerlen, Netherlands), which came in liquid form, was added at the 0.05% v/v dosage recommended by the manufacturer; and Proctase (Meiji Seika Pharma Co., Ltd., Tokyo, Japan), which came in powder form, was added directly at 30 mg/L. Protease additions were made immediately before heating. Based on a combination of results from an initial screening trial (conducted in triplicate on 200 mL aliquots of retentate, Supplementary Figure 1) and the commercial availability of each enzyme, DSM was selected for the larger scale treatments.

### Wine Treatments

**FIGURE 1:**
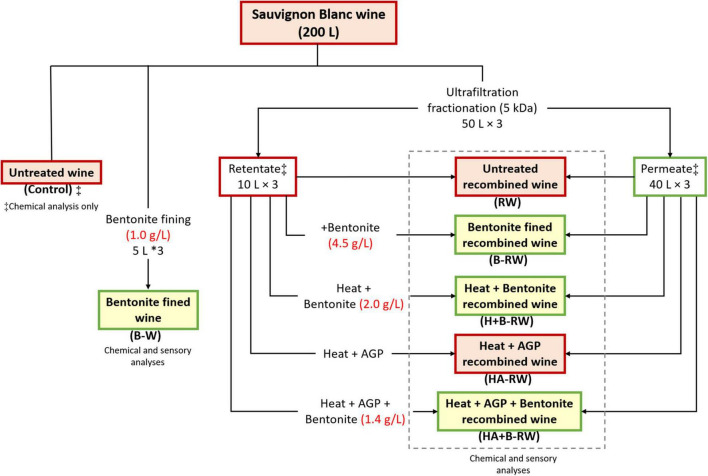
Flowchart of Sauvignon Blanc wine treatments and codes. Retentate heat treatment was 10 min at 62°C and Aspergillopepsin (AGP) protease dosage was 0.05% v/v DSM, if applied. Red boxes represent samples that were heat unstable (ΔNTU > 2), while samples in green boxes were heat stable (ΔNTU < 2). ^‡^Denotes samples that were subject to chemical analysis only. Chemical and sensory analyses were performed on all other wine samples.

Sauvignon Blanc wine (SAB) was fractionated by ultrafiltration (UF), followed by heat, protease and/or bentonite treatments (in triplicate), as shown in Figure 1 and described below.

Ultrafiltration (UF) of SAB wine (50 L, in triplicate) was carried out with a commercial crossflow membrane filtration system (VAF Memstar, Nuriootpa, SA, Australia) equipped with a 5 kDa (nominal MWCO) spiral-wound polyethersulfone (PES) membrane (surface area of 6.4 m^2^). The transmembrane pressure was controlled at 5–7 bar throughout treatment to generate a permeate flow (flux) of ∼2–3 L/min. Immediately prior to fractionation, the UF system was filled with nitrogen and dry ice was added to the retentate and permeate receival tanks, to prevent oxidation. UF generated 40 L of permeate and 10 L of retentate, with fractionation completed within ∼20 min; the 80% degree of permeation reflected the practical limit that could be replicated with the equipment available. The UF system was flushed with water between replicate UF treatments. Permeate and retentate samples were stored at 0^°^C prior to heat stabilization and/or recombination (specifically blending at 1:4 parts retentate:permeate).

Stabilization treatments were subsequently carried out on each retentate sample to reduce their protein concentrations prior to recombination with permeate (as above). The retentate fraction was divided into five aliquots (2 L each) and treatments were applied comprising (i) bentonite fining, (ii) heating and bentonite fining, (iii) heating with protease, and (iv) heating with protease followed by bentonite fining (Figure 1). Heat and/or protease treatments were performed in triplicate with a purpose-built heating unit, as described previously (9). Briefly, retentate (2 L) was heated at 62C° for 10 min, with or without the addition of AGP. After cooling, 50 mL of supernatant from each of the treated retentate samples was subsampled for macromolecule analysis. Heat stability tests were conducted on the retentate, and bentonite was added at the dose rate required to achieve heat stability. Prior to recombination with permeate (at a 1:4 volume ratio), retentate samples were stored at 0^°^C (∼3 days) before racking off lees. These treatments generated (i) bentonite fined wine (B-RW), (ii) heat and bentonite fined recombined wine (H+B-RW), (iii) heat with protease recombined wine (HA-RW), and (iv) heat with protease and bentonite fined recombined wine (HA+B-RW), respectively. Additionally, a treatment involving immediate blending of untreated retentate with permeate was included, producing (untreated) recombined wine (RW) samples, to assess the impact of UF treatment alone on wine chemical and sensory profiles. The codes and treatments for each wine are summarized in Figure 1. Samples (100 mL) of wine (untreated, bentonite treated, and recombined), retentate (before and after treatment), and permeate were collected for compositional analysis.

After heat stabilization and recombination, free SO_2_ levels were adjusted to ∼20 mg/L (by addition of potassium metabisulfite) prior to bottling in 750 mL glass bottles (Plastene, Adelaide, SA, Australia) with Saranex lined Novatwist closures (Plastene). Untreated SAB wine (10 L) was also bottled as a negative control (for chemical analysis only), while wine (5 L) treated only with bentonite (B-W, 1 g/L) was bottled (following SO_2_ adjustment, as above) for use as an industry-standard positive control. Bottled wines were cellared at 18^°^C until needed for chemical and sensory analyses (within 4 months).

### Chemical Analysis

#### Heat Stability

Heat stability tests were carried out on wine, permeate and retentate samples (50 mL) as previously described (17). Samples were considered to be heat stable when the change in turbidity (ΔNTU) before and after heating and cooling (2 h at 80^°^C, then 3 h at 20^°^C) was < 2 NTU, as measured using a turbidimeter (2100Qis, Hach Pacific, Dandenong South, Vic., Australia).

#### Basic Wine Chemistry

Measurement of pH, titratable acidity (TA), alcohol (% alcohol by volume, abv), free and total SO_2_, glucose and fructose (i.e., residual sugars), malic acid, and volatile acidity was performed using a Foss WineScan analyzer (Mulgrave, Vic., Australia); while total phenolics (A_280_ – 4), relative brown color (A_420_), flavonoids [A_280_ – 4] – [0.66 × (A_320_ – 1.4)], and total hydroxycinnamates [(A_320_ – 1.4)/0.9] × 10 were measured by UV-Vis spectrometry. Wine color was analyzed according to *OIV* CIELab method (OIV-MA-AS2-11) using 1 mm pathway cuvettes in a Cintra 4040 spectrometer (GBC Scientific Equipment, Braeside, Vic., Australia).

#### Wine Macromolecules

Wine macromolecules, including haze-forming proteins, total protein and polysaccharides, were measured in each permeate, retentate, and wine sample. Haze-forming proteins, specifically thaumatin-like proteins (TLPs) and chitinases, were quantified by HPLC against an external thaumatin standard curve (Sigma-Aldrich, Castle Hill, NSW, Australia), as previously reported (9). Results are expressed as mg/L of thaumatin equivalents. Total protein composition was determined using sodium dodecyl sulfate polyacrylamide gel electrophoresis (SDS-PAGE), as described previously (9). Polysaccharide composition was determined using size exclusion HPLC using a 50 kDa standard curve, as reported previously (18). Results are reported as mg/L of 50 kDa Pullulan standard equivalents (kit P-82 from Shodex, Showa Denko K.K., Tokyo, Japan).

#### Wine Volatile Compounds

The volatile profiles of all wine samples were determined using established stable isotope dilution analysis methods (19–21), to assess the impact of UF/heat/protease treatments on the volatile composition of wine, and specifically, the formation of any chemical markers of oxidation. Fermentation esters, alcohols, acids and acetates were determined by GC-MS (21), while oxidation aldehydes (methionol, methional, maltol, 2-methylbutanal, 3-methylbutanal, nonenal, hexanal, hexenal, heptenal, bendaldehyde, octenal, and phenylacetaldehyde) were measured by GC-MS/MS (20). Polyfunctional thiols were measured by HPLC-MS/MS (19). The preparation/origin of isotopically labeled internal standards, method validation and instrument operating conditions were as previously reported.

### Sensory Analysis

The wines from each treatment were evaluated by five sensory experts (each with > 10 years of wine sensory experience) to establish there were no obvious sensory differences between replicates. Wine replicates were then blended prior to formal sensory analysis. Informed consent was obtained from sensory panelists and this study was approved by the Human Research Ethics Committee of the University of Adelaide (approval number: H-2019-073). Sensory analysis was not performed on the untreated wine (since it was not protein stable).

#### Quality Ratings

A panel of wine experts (*n* = 8, 3 female and 5 male) comprising winemakers and wine show judges who met the definition of experts as previously described (22), was convened. Panelists were asked to rate the quality of each treated wine using the 20-point scoring system employed in the Australian wine industry, including at wine shows (23). Demographics and tasting notes were also collected, along with responses to questions specifically asking if any oxidative or cooked characters were perceived. During the tasting, chilled wine samples (10^°^C, 25 mL) were served in 4 digit-coded, clear 215 mL stemmed wine glasses (Viticole IXL 5, covered with plastic lids), using a randomized presentation order. Water and plain crackers were provided as palate cleansers. Expert panel tasting was held in the Wine Experience Room at the Jacob’s Creek Visitor Centre (Barossa Valley, SA, Australia).

#### Descriptive Sensory Profiling

The sensory profiles of wines were characterized by a panel of non-expert consumers using the Rate-All-That-Apply (RATA) method (24). The panelists (*n* = 54, 36 female and 18 male, aged 18–67 years), all of whom were regular wine consumers, were recruited *via* email from an existing database. Before the tasting commenced, the use of the sensory booths and RATA procedure (including a list of attributes comprising varietal descriptors for Sauvignon Blanc, as well as attributes associated with oxidation, Supplementary Table 1), were explained to panelists. RATA assessments were conducted in a single session in sensory booths with controlled environmental conditions (i.e., lighting and a constant 22–23°C temperature). As for the expert panel tasting, chilled wine samples (10^°^C, 25 mL) were served in 4 digit-coded, clear 215 mL stemmed wine glasses (Viticole IXL 5, covered with plastic lids), using a randomized presentation order. Panelists rated the intensity of each sensory attribute using line scales, where 0 = “not perceived”, 1 = “extremely low”, 4 = “moderate” and 7 = “extremely high”. A 1 min break was enforced between samples, which were presented one at a time, and water and plain crackers were provided for palate cleansing. Data were collected using Red Jade software (Redwood Shores, CA, United States).

### Data Analysis

Wine compositional data were analyzed by one-way ANOVA using GraphPad Prism 8 (San Diego, CA, United States). Mean comparisons were performed by Tukey’s honestly significant difference multiple comparison test at *P* < 0.05. Wine sensory data were analyzed by two-way ANOVA using participants as a random factor and wines as a fixed factor, using Addinsoft XLSTAT (2020.5.1, New York, NY, United States). Partial least squares regression (PLSR) was performed using the Unscrambler X (version 10.3, CAMO Process, Oslo, Norway) and cross-validated using a random test to correlate wine chemical composition (X variables) with sensory (Y variables).

## Results and Discussion

### Impact of Protein Stabilization Treatments on Heat Stability, Color, and Macromolecule Composition of Retentate

Ultrafiltration (UF) of the SAB wine generated heat stable permeate (ΔNTU = 0.2 ± 0.1) and heat unstable retentate (ΔNTU = 115.5 ± 4.8). This enabled targeted treatment of the proteins concentrated in the retentate (20% of the initial wine volume), instead of treatment of the whole wine. DSM was chosen for subsequent treatments as it is currently readily available on the market and the extent of protein removal was similar to the previously reported product, Proctase (Supplementary Figure 1). Neither heat treatment alone nor heat treatment with AGP addition removed all haze-forming proteins (Supplementary Figure 1). Therefore, bentonite fining (BF) was performed on (i) untreated retentate, (ii) heat treated retentate, and (iii) retentate heated with AGP to achieve heat stability in recombined wine. Combined heat and protease treatment of the retentate substantially reduced the amount of bentonite required to achieve protein stabilization of the recombined wine compared to bentonite fining alone (1.4 and 4.5 g/L, respectively, Figure 1). UF effectively concentrated all proteins, enabling their targeted removal from the retentate fraction, thereby reducing the overall bentonite requirement compared with fining the whole wine; i.e., from 1.0 g/L for whole wine to 0.9, 0.4, and 0.28 g/L (as wine volumes) for bentonite, heat and bentonite, and heat with AGP and bentonite treatments of retentate, respectively.

**FIGURE 2:**
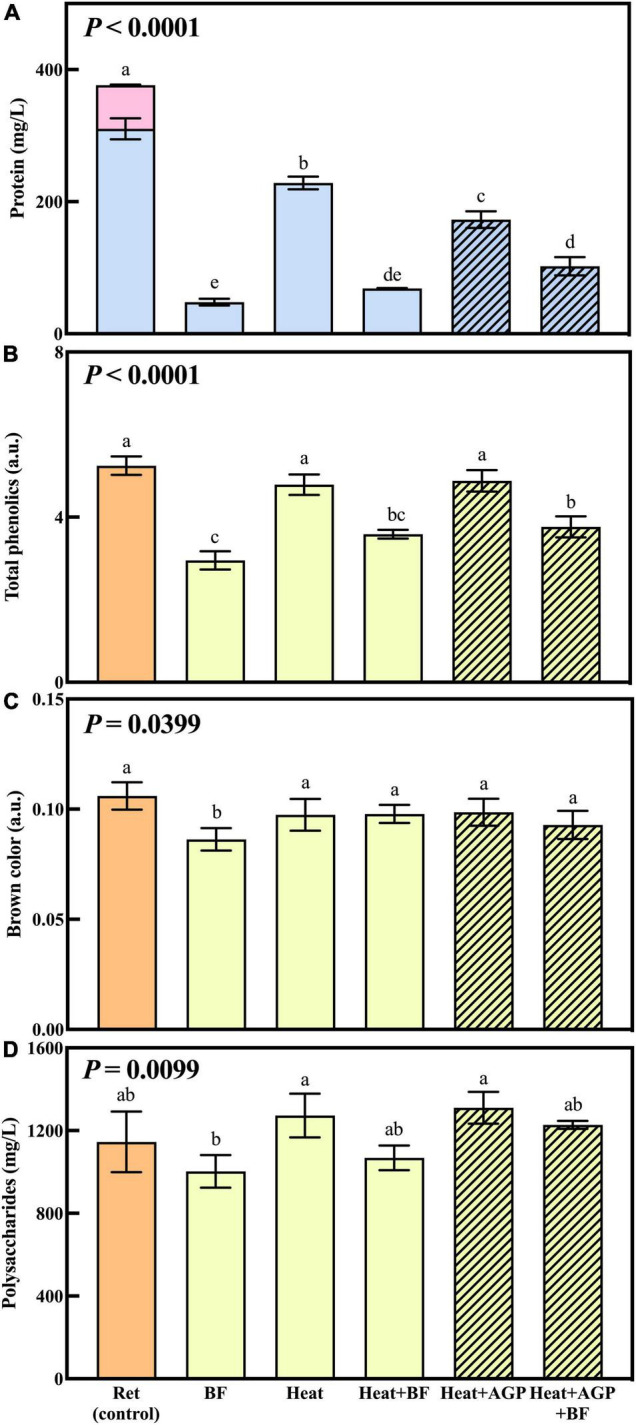
Haze-forming protein composition (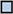 TLPs and 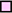 chitinases) **(A)**, total phenolics **(B)**, brown color **(C)**, and polysaccharide concentrations **(D)** of untreated retentate (control) and retentate treated *via* bentonite fining (BF) and/or heating (10 min at 62°C), with or without AGP (0.05% v/v DSM) addition. Shading denotes retentate treated with AGP. Data are means of three replicates (± standard error), with the exception of polysaccharide concentrations of untreated retentate, for which values are means of two replicates. Different letters indicate statistically significant differences (one-way ANOVA, Tukey’s HSD, *P* < 0.05).

The effect of UF, heat and/or protease treatment on wine macromolecules and color is shown in Figure 2. Figure 2A illustrates the composition and concentration of haze-forming proteins in retentate samples before (control) and after the treatments detailed in Table 1. Protein concentrations in treated retentate samples were significantly lower than untreated retentate. Heating removed 39% of haze-forming proteins in retentate, while the addition of AGP during heating removed a further 15% of haze-forming proteins (54% overall). The protein removal achieved with heat and AGP in this study indicated enhanced protein degradation with protease addition, whereas our previous study reported no significant differences between heat treatments with or without the addition of protease (9). The greater efficacy of protease performance observed in the current study may be due to treatment on a larger scale with the potential for longer heating and cooling times due to the increased treatment volumes, as well as the inherent variation in activity of enzymes from different sources and batches. TLPs are the most abundant proteins in white wine (25) and constituted the majority of haze-forming proteins observed in untreated retentate (control, Figure 2A). Chitinases were readily removed by all treatments (bentonite fining, heating and heating with AGP addition), in agreement with previous research that found chitinases are less heat stable, and more easily removed with bentonite (26, 27). The concentrations of haze-forming protein in heated and bentonite-fined retentate (Heat + BF) were not significantly different to either bentonite-fined (BF) retentate or retentate heated with AGP and then bentonite fined (Heat + AGP + BF), as expected, such that stable recombined wines contained similar levels of haze-forming proteins (Table 1). This targeted approach of fining only the macromolecule-rich fraction is more likely to reduce the impact of bentonite addition on wine volatile profiles and hence sensory properties.

**TABLE 1:** Basic chemistry, color, macromolecule composition, heat stability and quality ratings of untreated wine (Control), wine treated *via* bentonite fining (B-W) as a positive control, and recombined wines (RW) following ultrafiltration/heat/protease treatments; B denotes bentonite addition; H denotes heating (10 min at 62°C); A denotes Aspergillopepsin enzyme addition (DSM 0.05% v/v).

Parameter	Control	B-W	RW	B-RW	H+B-RW	HA-RW	HA+B-RW	*P*-value
pH	3.2 ± 0.01b	3.2 ± 0.01a	3.2 ± 0.00a	3.2 ± 0.01a	3.2 ± 0.00a	3.2 ± 0.01a	3.2 ± 0.01a	*0.0001*
TA (g/L)	6.0 ± 0.06a	6.0 ± 0.00a	6.0 ± 0.06a	5.9 ± 0.00b	6.0 ± 0.00a	6.0 ± 0.00a	6.0 ± 0.00a	*0.0022*
Alcohol (% v/v)	11.8 ± 0.00a	11.6 ± 0.00b	11.6 ± 0.00b	11.4 ± 0.00d	11.5 ± 0.00c	11.6 ± 0.00b	11.5 ± 0.06c	<*0.0001*
Free SO_2_ (mg/L)	23.3 ± 0.6	24.7 ± 7.0	20.7 ± 0.6	20.3 ± 2.3	27.3 ± 3.8	20.0 ± 1.0	22.0 ± 0.0	*ns*
Total SO_2_ (mg/L)	116.0 ± 1.7	116.3 ± 11.5	108.7 ± 1.5	108.3 ± 4.0	120.7 ± 5.8	108.3 ± 1.2	111.7 ± 0.6	*ns*
Residual sugars (g/L)	1.3 ± 0.06a	1.1 ± 0.06bc	1.1 ± 0.00bc	1.0 ± 0.00c	1.0 ± 0.06bc	1.2 ± 0.06b	1.0 ± 0.06bc	<*0.0001*
Malic acid (g/L)	1.3 ± 0.01b	1.3 ± 0.01b	1.3 ± 0.01a	1.3 ± 0.02a	1.3 ± 0.01a	1.3 ± 0.01a	1.3 ± 0.01a	*0.0006*
Total phenolics (a.u.)	3.2 ± 0.01a	2.1 ± 0.02c	2.6 ± 0.15b	2.1 ± 0.15c	2.2 ± 0.2c	2.4 ± 0.15bc	2.3 ± 0.13bc	<*0.0001*
Flavonoids (a.u.)	1.5 ± 0.01a	0.5 ± 0.02d	1.1 ± 0.07b	0.7 ± 0.08c	0.8 ± 0.09c	1.0 ± 0.08b	0.9 ± 0.07bc	<*0.0001*
Hydroxycinnamates (a.u.)	29.0 ± 0.00a	27.0 ± 0.00abc	24.3 ± 1.16c	23.0 ± 1.00c	23.3 ± 1.16c	23.7 ± 1.53c	23.3 ± 1.16c	<*0.0001*
Brown color (a.u.)	0.06 ± 0.00a	0.05 ± 0.01ab	0.05 ± 0.00ab	0.04 ± 0.01b	0.05 ± 0.01b	0.05 ± 0.00ab	0.05 ± 0.01b	*0.0083*
Protein (mg/L)	84.2 ± 1.0a	11.2 ± 2.2c	76.0 ± 2.9a	2.7 ± 0.1c	4.3 ± 0.7c	34.6 ± 8.8b	13.3 ± 1.7c	<*0.0001*
ΔNTU	29 ± 1.9a	1.0 ± 0.4d	22 ± 1.1b	1.2 ± 0.4d	1.0 ± 0.2d	4.4 ± 0.2c	1.9 ± 1.1cd*	<*0.0001*
Polysaccharides (mg/L)	213.5 ± 65.7	230.3 ± 15.5	250.3 ± 20.4	205.0 ± 10.9	208.3 ± 20.1	241.4 ± 15.8	181.3 ± 7.2	*ns*
Quality rating (/20)	–	14.8 ± 1.5	14.5 ± 1.7	14.4 ± 2.0	14.5 ± 1.1	14.6 ± 1.3	14.5 ± 2.6	*ns*

Data are means of three replicates (± standard deviation). Means followed by different letters are statistically significant (one-way ANOVA, Tukey’s HSD, P < 0.05); ns, not significant. Wine is considered to be heat stable when ΔNTU < 2. *Indicates one of the replicates was not stable (ΔNTU > 2). The italicised values are P-values.

The total phenolics, brown color and polysaccharide concentrations in the retentate increased significantly with UF (by 1.7, 2.2, and 4.8-fold, respectively) compared to the control (data not shown). The permeate comprised significantly lower total phenolics and brown coloration, with no detectable polysaccharides (data not shown). This was in agreement with the previous study (9) that reported UF affected the overall macromolecule composition of wine; large wine soluble molecules, i.e., some phenolics and all polysaccharides, were rejected by UF membranes (9).

Analyses were repeated on treated retentate samples to study the impact of protein stabilization treatments on total phenolics, brown color and polysaccharides, before they were blended with their corresponding permeate to generate recombined wine (Figures 2B–D). Heating retentate with or without AGP addition did not significantly affect the total phenolics compared to the control (Figure 2B). However, bentonite addition gave significantly lower total phenolic levels compared with the control and heated retentates, consistent with findings from a previous study (28). In white wine, brown color is regarded as an important indicator of wine oxidation or age (29, 30, 31). Brown color was not enhanced by any of the heat treatments (Figure 2C), indicating that the heating condition employed (62°C for 10 min) did not introduce phenolic oxidation (30). Only the bentonite-fined retentate had a significantly lower brown color (Figure 2C), that was attributed to the significant phenolic removal (Figure 2B), which most likely removed colored phenolic pigments (31). Overall, the addition of bentonite to retentate significantly reduced total phenolics, brown color and polysaccharides (Figures 2B–D), along with protein removal. This reflects the non-specific binding performance of bentonite (5, 28) compared to the targeted protein degradation achieved *via* heat treatment (with and without AGP). Given the importance of wine macromolecules to organoleptic qualities (32), these compositional changes might reasonably be expected to influence wine sensory profiles.

### Impact of Protein Stabilization Treatments on Wine Chemistry and Volatile Composition

#### Wine Chemical Composition

The composition of recombined wine (RW) without any retentate treatment was compared with that of the untreated wine (control) and the bentonite-fined wine (B-W) as a positive control (Table 1). UF did not significantly affect free and total SO_2_ or polysaccharide concentrations, but significant changes in pH, TA, alcohol, residual sugars, malic acid, brown color (as A420) and hydroxycinnamates were observed between treated wines and the untreated control, albeit they were not considered substantial from a winemaking perspective. The other recombined wines gave similar results—especially compared with the positive control, B-W. The low TA, alcohol and residual sugar levels observed in B-RW may reflect dilution from bentonite slurry addition. Volatile acidity (as acetic acid) was less than 0.25 g/L for all wine samples (data not shown) and well below the maximum acceptable level of 1.2 g/L (33).

The control wine had the highest total phenolics content and all treatments yielded significantly lower wine total phenolics (Table 1), in accordance with results for retentate (Figure 2B). A 19% decrease in total phenolics was observed in RW due to UF fractionation alone (Table 1), which may relate to adsorption of phenolics to the PES membrane (34–36). Interactions between proteins and phenolics (protein-phenolic binding) may also facilitate their removal from wine (35–37). As expected, the RW and control had similar protein concentrations since no stabilization measures were applied to either wine. However, despite being unstable, the lower haze potential (ΔNTU = 22) of RW compared with the control (ΔNTU = 29) can be attributed to the decrease in phenolics, which are associated with crosslinking during haze formation (3). Bentonite addition to either wine or retentate resulted in a greater loss of phenolics. The variation in wine phenolics after stabilization treatments are more likely to be attributed to flavonoids rather than hydroxycinnamates (Table 1), due to complexation with proteins and/or interactions with the membrane (38) resulting in their concentration in retentate. Unlike the brown color results for retentate (Figure 2C), UF and heating with protease did not significantly affect the brown color of recombined wine compared with control and B-W wine. However, recombined wines derived from retentate treated with bentonite (i.e., B-RW, H+B-RW, and HA+B-RW) had significantly lower brown color measurements (Table 1). Analysis of wine color using CIELab confirmed UF/heat/protease treatments did not introduce color changes in recombined wines (Supplementary Table 2).

Traditional fining with 1 g/L of bentonite (minimal dosage to stabilize wine) removed haze-forming proteins and gave heat stable wine B-W (ΔNTU = 1 ± 0.4). Bentonite fining of retentate (either treated or untreated retentate) similarly reduced haze-forming proteins, thus B-RW, H+B-RW, and HA+B-RW were all heat stable (Table 1). However, one of the HA+B-RW replicates failed the heat stability test after cooling (ΔNTU = 3.2), which suggests bentonite fining is not always reliable, in agreement with anecdotal evidence from winemakers. SDS-PAGE results (Supplementary Figure 2) demonstrated that bentonite removed all wine proteins including lipid transfer proteins (LTPs) and invertases which do not participate in haze-formation (7, 28), whereas AGP selectively removed those proteins associated with low conformational stability and haze (i.e., β-glucanase, chitinases and some TLPs) (39–41). In addition, SDS-PAGE confirmed AGP was not present in treated wines. More than half (59%) of the haze-forming proteins were removed from HA-RW compared with the control. After stabilization, HA-RW contained a higher concentration of TLPs (34.6 mg/L) than bentonite treated wines, and subsequently failed the heat stability test. However, the heat test was conducted at a temperature (80^°^C) leading to the precipitation of all wine proteins, even those (such as invertase) that are known to be heat stable and that would not precipitate in the bottle (26, 40, 42). The residual haze in the HA-RW after the 80^°^C heat test therefore seems likely to be due to precipitation of proteins that would not form haze in bottled wine. Thus, the more stable TLPs remaining in the HA-RW may not develop any haze during cellar aging; but to validate this hypothesis, shelf-life studies are required (4, 26, 39, 43, 44). Wine protein and polysaccharide compositions play important roles in foamability, particularly for sparkling wines (45). Bentonite treatment leads to a loss of wine foamability due to the removal of grape proteins. The important role of invertase in foam formation and stability has previously been identified (46, 47). Thus, after stabilization the preservation of invertase and some pathogenesis-related proteins without bentonite addition may contribute to better foam quality in sparkling wine, therefore potentially favoring this novel UF/heating/protease treatment over bentonite fining.

#### Influence of Protein Stabilization Treatments on Wine Volatile Composition

Sauvignon Blanc (*Vitis vinifera*) is an important white grape cultivar and it has become popular in New World wine producing countries such as Australia and New Zealand (48). Wine made from this variety can exhibit both grassy/boxwood and tropical characters (48, 49). Volatiles, including esters, methoxypyrazines and thiols, are important contributors to the aromas and flavors of Sauvignon Blanc wine (50). Many previous studies have used Sauvignon Blanc due to its high protein content, which makes it a suitable candidate for studying white wine protein stabilization (40–42, 51–53).

**FIGURE 3:**
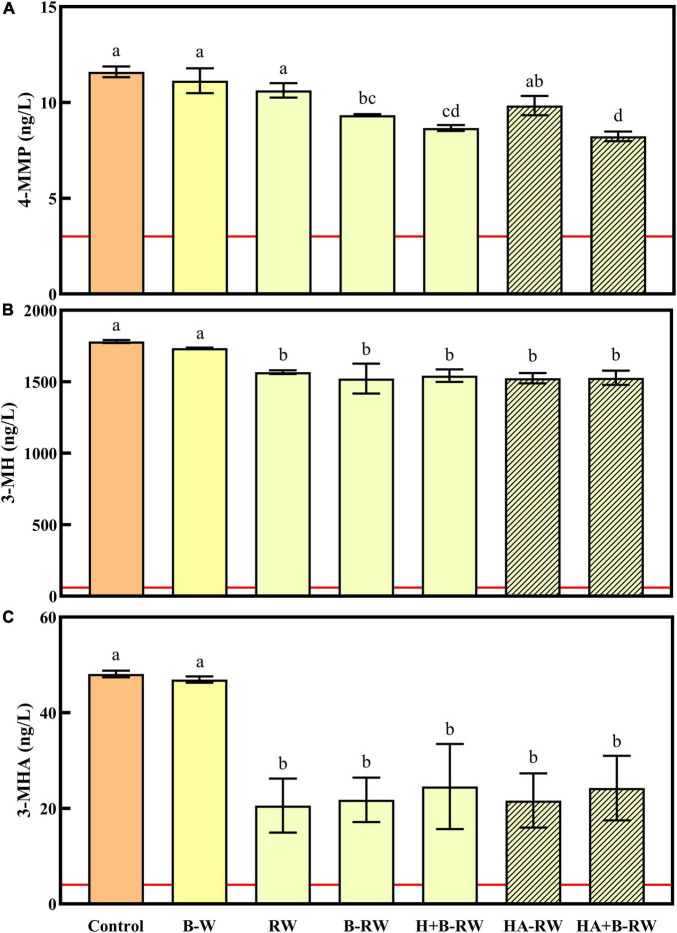
Concentrations of **(A)** 4-MMP, **(B)** 3-MH, and **(C)** 3-MHA in untreated wine (Control), wine treated *via* bentonite fining (B-W) as a positive control, and recombined wines (RW) following ultrafiltration/heat/protease treatments; B denotes bentonite addition; H denotes heating (10 min at 62°C); A denotes Aspergillopepsin enzyme addition (DSM 0.05% v/v). The odor detection thresholds of each varietal thiol are marked as red lines. Data are means of three replicates (± standard error). Different letters indicate statistically significant differences (one-way ANOVA, Tukey’s HSD, *P* < 0.05).

Varietal and fermentation volatiles were quantified (19, 21) in control and treated wines (Table 2, Supplementary Table 3, and Figure 3). Of the 37 volatiles measured, 27 were significantly different amongst control and treated wines, the majority of which were ethyl and acetate esters and alcohols derived from fruit and/or yeast during alcoholic fermentation, typically associated with fruity and floral notes in wine (21). Other classes of compounds included monoterpenes that impart positive floral characters, volatile fatty acids that tend to exhibit negative sensory attributes, and potent sulfur compounds that contribute the key varietal attributes associated with SAB wines.

**TABLE 2:** Concentrations of volatile compounds determined in untreated wine (Control), wine treated *via* bentonite fining (B-W) as a positive control, and recombined wines (RW) following ultrafiltration/heat/protease treatments; B denotes bentonite addition; H denotes heating (10 min at 62°C); A denotes Aspergillopepsin enzyme addition (DSM 0.05% v/v).

Compound	Control	B-W	RW	B-RW	H+B-RW	HA-RW	HA+B-RW	*P*-value
***Ethyl esters***								
Ethyl propanoate	93.9ab	97.2ab	89.4abc	86.9bc	81.8c	81.4c	97.7a	*0.0203*
Ethyl-2-methylproanoate	82.5	83.3	82.7	79.0	78.0	80.7	81	*ns*
**Ethyl butanoate**	300.8	306.1	282.6	269.9	260.6	259.7	307.9	*ns*
**Ethyl 2-methylbutanoate**	4.5	4.5	4.2	3.9	3.7	4.0	4.3	*ns*
**Ethyl 3-methylbutanoate**	5.0	5.0	4.7	4.6	4.4	4.6	4.6	*ns*
**Ethyl hexanoate**	784.7a	765.3a	717.9a	668.9ab	577.8b	562.4b	724.9a	*0.0315*
Ethyl lactate (mg/L)	8.8b	8.7bc	8.8b	8.5bc	8.4c	8.6bc	10.2a	<*0.05*
**Ethyl octanoate**	479.5a	447.9a	313.5b	308.1b	206c	275.6bc	315.4b	*0.0014*
Ethyl decanoate	103.1a	91.3b	75.1c	71.6d	69.1d	70.0d	71.7d	<*0.05*
Diethyl succinate	561.1a	511.6a	516.5a	440.2ab	317.6c	329.3bc	482.2a	*0.0036*
Ethyl 2-phenylacetate	1.8a	1.8a	1.7b	1.7b	1.7b	1.7b	1.7b	*0.0002*
***Acetate esters***								
**Ethyl acetate (mg/L)**	47.1ab	47.1ab	43.4bc	42.0bc	41.3c	40.7c	49.7a	*0.0149*
**3-Methylbutyl acetate (mg/L)**	2.3	2.4	2.1	2.0	2.0	1.9	2.4	*ns*
Hexyl acetate	230.2c	223.9c	297.8b	273.9b	271.3b	280.1b	329.6a	<*0.05*
**2-Phenylethyl acetate**	292.8a	282.8a	250b	248.7b	246.3b	248.7b	278.1ab	*0.0408*
***Alcohols***								
**1-Propanol (mg/L)**	25.4ab	25.8a	24.0bc	23.5c	22.9c	23.3c	26.7a	*0.0021*
2-Methyl-1-propanol (mg/L)	15.1bc	15.2b	14.5bcd	14.2d	14.0d	14.3cd	16.3a	*0.0004*
1-Butanol	592.6bc	623.9b	569.4cd	573.1cd	552.8d	573.4cd	670.2a	<*0.05*
**3-Methyl-1-butanol (mg/L)**	107.3bc	109.5b	106.6bc	103.8c	102.3c	104.9bc	121.9a	<*0.05*
1-Hexanol (mg/L)	2.3bc	2.3b	2.2bc	2.2bc	2.1c	2.2bc	2.5a	*0.002*
3-Octanol	6.7	6.7	6.7	6.7	6.7	6.7	6.7	*ns*
2-Ethyl-1-hexanol	5.6	5.3	7.2	6.2	6.5	6.8	7.7	*ns*
**1-Octanol**	10.7abc	10.9ab	10.5bc	10.1bc	9.8c	9.9c	11.7a	*0.0081*
Benzyl alcohol	137.5a	136.4a	127.3b	122.3b	121.9b	123.1b	137.9a	*0.0024*
2-Phenylethanol (mg/L)	13.4a	13.2ab	12.2bc	11.7c	11.7c	12.1bc	13.6a	*0.0079*
***Isoprenoids***								
Linalool	9.6c	9.9c	10.5bc	11.3ab	9.7c	12.1a	11.7ab	*0.0034*
α-Terpineol	19.8a	19.5a	19.1ab	18.5c	18.6bc	18.1c	19.4a	*0.0005*
***Acids***								
Acetic acid (mg/L)	146.5ab	148.7ab	139.5b	144.4b	139.7b	136.9b	160.5a	*0.0373*
Isobutanoic acid	429.9bc	431.8b	407.8bc	386.2c	402.3bc	409.9bc	483.8a	*0.0047*
Butanoic acid (mg/L)	1.6b	1.6bc	1.5bcd	1.5d	1.5d	1.5cd	1.7a	*0.0002*
3-Methylbutanoic acid	358.5b	349.7bc	347.9bcd	338.6d	338.2d	339.3cd	377.7a	<*0.05*
**Hexanoic acid (mg/L)**	6.5	6.7	6.3	6.1	6.1	6.3	7.2	*ns*
**Octanoic acid (mg/L)**	22.1	21.4	13.7	11.4	12.7	12.1	15.8	*ns*

*Concentrations are presented in μg/L except where otherwise noted. Volatiles present at supra-threshold concentrations are shown in bold/italics. Data are means from three replicates (n = 3), except for Control wines which are means from technical duplicates. Means followed by different letters are statistically significant (one-way ANOVA, Tukey’s HSD, P < 0.05); ns, not significant. The italicised values are P-values.*

The volatiles present at supra-threshold concentrations (shown in bold in Table 2) that differed among wine samples included ethyl hexanoate, ethyl octanoate, ethyl acetate, 2-phenylethyl acetate, 1-propanol, 3-methyl-1-butanol, and 1-octanol. These volatiles are generally considered to impart “chemical” aromas, with the exception of ethyl hexanoate, ethyl octanoate and 2-phenylethyl acetate which impart “fruity” aroma (21). Other volatiles present at concentrations below their corresponding aroma detection thresholds would be less likely to make direct contributions to wine sensory profiles.

The negative control and B-W had similar volatile compositions (Table 2), suggesting that in the current study, bentonite treatment did not strip significant quantities of the volatile compounds measured. B-W did have a lower level of ethyl decanoate, which agreed with previous studies that report the loss of this ester due to protein removal with bentonite addition (5, 25). The lower volatile concentrations observed for RW (compared with the control), particularly for ethyl octanoate, ethyl decanoate, ethyl 2-phenylacetate, 2-phenylethyl acetate, benzyl alcohol and 2-phenylethanol, suggest that UF treatment alone resulted in the loss of some wine volatiles. Although it remained below its reported detection threshold concentration, UF treatment increased levels of the yeast-derived volatile, hexyl acetate (typically described as “fruity” and “floral”), in all recombined wines, which may reflect esterification of C6 precursors (54). Addition of bentonite to retentate led to volatile losses in B-RW similar to those observed in RW (Table 2), with a greater loss of α-terpineol.

Heating retentate with AGP resulted in small, but significant decreases in some of the fruity volatile compounds present in HA-RW, including ethyl hexanoate, ethyl octanoate and 2-phenylethyl acetate. Some of the lowest concentrations of volatile compounds were observed in H+B-RW, but it’s unclear if volatile losses were attributable to UF, heat or bentonite treatment, given similar losses were not observed in HA+B-RW. Indeed, HA+B-RW had comparable or even higher concentrations of most volatiles than thecontrol wine.

The monoterpenes, linalool and α-terpineol, were identified in all wines, and are responsible for floral characters in wine (21). Treated recombined wines (B-RW, HA-RW, and HA+B-RW) had significantly higher levels of linalool than the negative control, B-W and H+B-RW. Whereas HA+B-RW maintained its α-terpineol concentration, other wines derived from stabilization treatments (B-RW, H+B-RW, and HA-RW) had significantly lower α-terpineol levels (Table 2). The varied levels of linalool and α-terpineol in treated recombined wines suggest they have gone through transformations after treatments (55).

Several volatile fatty acids were also detected in control and treated wines. HA+B-RW had significantly higher levels of isobutanoic acid, butanoic acid and 3-methylbutanoic acid, as compared to the control and other treated wines, however, these compounds were all below their corresponding detection threshold concentrations (56).

Volatile sulfur compounds contribute to the signature tropical fruit characters of Sauvignon Blanc wine, particularly the polyfunctional varietal thiols: 4-mercapto-4-methylpentan-2-one (4-MMP), 3-mercaptohexan-1-ol (3-MH) and 3-mercaptohexyl acetate (3-MHA) (19, 57). In the current study, 4-MMP, 3-MH and 3-MHA were present at concentrations above their reported detection thresholds (3, 60, and 4 ng/L, respectively) in all wine samples (Figure 3). Benzyl mercaptan was also measured but was not detected in any wines (i.e., concentrations were < 2.5 ng/L). Bentonite fining did not affect the varietal thiol concentrations of the SAB wine, but UF treatment of wine resulted in 12–15 and 49–57% losses of 3-MH and 3-MHA, respectively (Figures 3B,C), presumably due to polyphenol oxidation during UF fractionation (57), which may have inadvertently caused some aeration. The increased loss of 3-MHA likely reflects its susceptibility to hydrolysis (57). Neither UF fractionation nor heating retentate with AGP (RW and HA-RW) affected 4-MMP concentrations. However, the other stabilization treatments applied to retentate [i.e., bentonite addition, and heating (with and without AGP) followed by bentonite addition] significantly reduced 4-MMP levels (Figure 3A). Although UF/heat/protease treatments had some impact on varietal thiol concentrations, as mentioned above, they remained well above detection thresholdsconcentrations.

Volatile compounds regarded as markers of oxidation were measured in control and treated wines to establish to what extent, if any, UF/heat/protease treatments might introduce oxidative characters to wine as a consequence of any aeration. Of the 15 oxidation volatiles quantified (Supplementary Table 4), only 2-methylbutanal, 3-methylbutanal, methional, 2-non-enal, and 2-phenylacetaldehyde were detected (in all wines) at concentrations above their respective reported aroma detection thresholds (20). Importantly, these compounds were observed at concentrations similar to or lower than those reported in previous studies (including analysis of commercial wines), as summarized in Supplementary Table 4. Additionally, three other volatiles [(*E*)-2-hexenal, maltol, and 5-methylfurfural] were not detected in any of the wine samples. UF treated recombined wine RW had similar levels of oxidative volatiles compared to the control and B-W (Supplementary Table 4). UF treatment and heating of retentate did not significantly change 3-methylbutanal, (*E*)-2-nonenal and 2-phenylacetaldehyde concentrations in wine. Results of 3-methylbutanal were much lower than previously measured in commercial wines (58). These results showed that UF/heat/protease treatments did not introduce oxidative characters to wine.

### Influence of Protein Stabilization Treatments on Wine Quality and Sensory Profiles

Wine quality was assessed by an expert panel of eight winemakers and there was no significant difference amongst quality ratings, which ranged from 14.4 to 14.8 (Table 1). Descriptors were collated from expert panelists’ tasting notes and largely comprised fruit attributes, with no obvious faults (e.g., oxidation) being flagged. However, the panel’s tasting notes did suggest HA-RW and H+B-RW exhibited riper fruit characters (“tropical”, “mango”, “stone fruit” and “melon”) and a slight “cooked fruit” character, and lacked floral notes relative to other wines.

**FIGURE 4:**
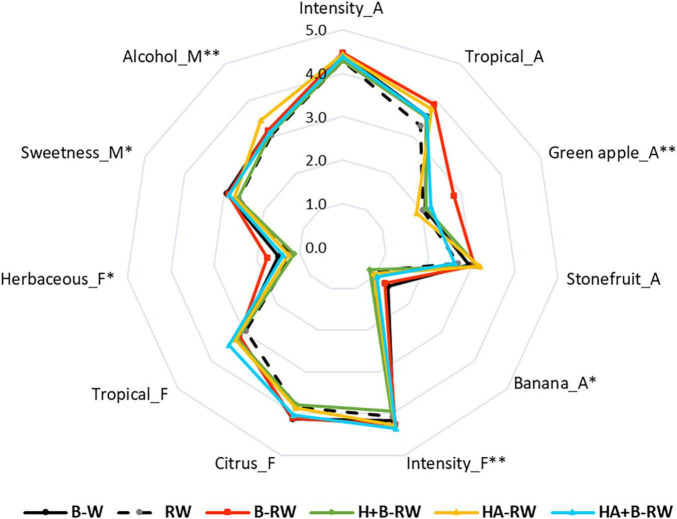
Sensory profiles of wine treated *via* bentonite fining (B-W), and recombined wines (RW) following ultrafiltration/heat/protease treatments; B denotes bentonite addition; H denotes heating (10 min at 62°C); A denotes Aspergillopepsin enzyme addition (DSM 0.05% v/v). A, F and M represent aroma, flavor and mouthfeel attributes, respectively. Results are means of panel rating (*n* = 54). ^**^ and *indicate statistical significance at *P* < 0.05 and *P* < 0.1 confidence levels.

The sensory profiles of treated SAB wines were determined using the Rate All That Apply (RATA) sensory analysis method (24). Of the 38 attributes assessed, panelists perceived only 3 sensory attributes to differ significantly (*P* < *0.05*) between wine treatments: green apple aroma, overall flavor intensity, and alcohol heat/warmth mouthfeel (Figure 4 and Supplementary Table 1). Overall flavor intensity was rated highest in HA+B-RW and lowest in H+B-RW, which agreed with the fermentation volatile data reported in “Influence of Protein Stabilization Treatments on Wine Volatile Composition.” However, recombined wines were not significantly different from B-W (the positive control). The enhanced green apple aroma was only perceived in B-RW, while the perception of alcohol heat was noted for HA-RW; albeit ratings for alcohol heat were not significantly different amongst heat-stable wines (Supplementary Table 1). Differences in the intensity of banana aroma, herbaceous flavor and sweetness were perceived at a lower level of confidence (i.e., at *P* < *0.1*). Overall aroma intensity was rated similarly across all wines. Citrus, tropical fruit, stone fruit, green apple, and floral attributes received relatively high ratings (both on the nose and the palate) by the panel (Supplementary Table 1), in agreement with the typical sensory profiles of Sauvignon Blanc wines (59).

**FIGURE 5:**
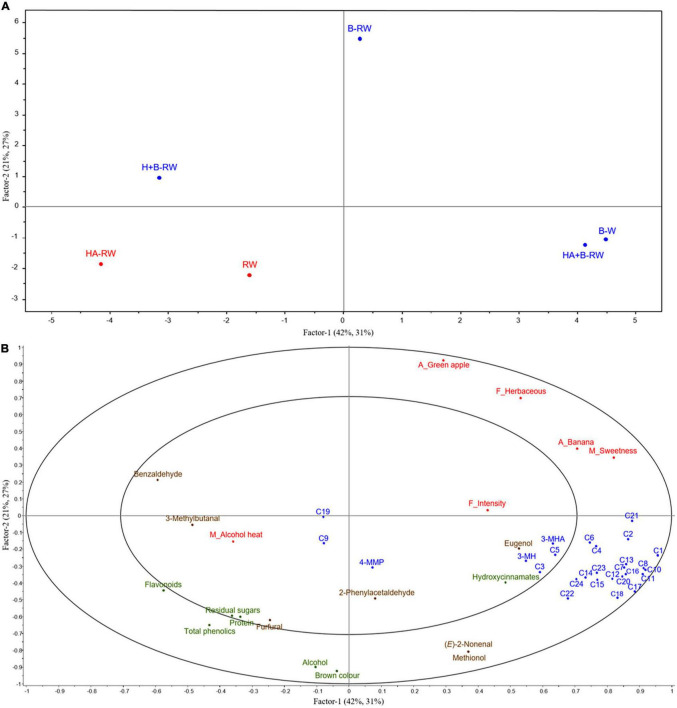
Correlations of chemical measurements (*P* < 0.05, X variables) and intensity ratings of statistically significant (*P* < 0.1) sensory attributes (Y variables) for wines using PLSR plots including **(A)** scores of wine samples: bentonite fining (B-W) as a positive control, and recombined wines (RW) following ultrafiltration/heat/protease treatments; B denotes bentonite addition; H denotes heating (10 min at 62°C); A denotes Aspergillopepsin enzyme addition (DSM 0.05% v/v). Red indicates unstable wines and blue indicates protein stable wines; and **(B)** X and Y loadings with 50% (inner) and 100% (outer) explained variance limits. X variables are in green (basic wine measurement), blue (varietal and fermentation volatiles), brown (oxidative volatiles) and Y variables in red. Codes for wine volatile compounds are as follows: C1, ethyl propanoate; C2, ethyl hexanoate; C3, ethyl lactate; C4, ethyl octanoate; C5, ethyl decanoate; C6, diethyl succinate; C7, ethyl 2-phenylacetate; C8, ethyl acetate; C9, hexyl acetate; C10, 2-phenylethyl acetate; C11, 1-propanol; C12, 2-methyl-1-propanol; C13, 1-butanol; C14, 3-methyl-1-butanol; C15, 1-hexanol; C16, 1-octanol; C17, benzyl alcohol; C18, 2-phenylethanol; C19, linalool; C20, α-terpineol; C21, acetic acid; C22, isobutanoic acid; C23, butanoic acid; C24, 3-methylbutanoic acid.

Partial least squares regression (PLSR) was performed to investigate the underlying relationship between wine composition (including volatile compounds) and sensory characteristics (21, 59). Statistically significant chemical markers (*P* < 0.05, X variables) and sensory attributes (*P* < 0.1, Y variables) were correlated against one another and plotted by PLSR (Figure 5). Factors 1 and 2 explained 58% of the sensory variation. The separation of wines was mainly attributed to the intensity of green apple and banana aroma, herbaceous flavors, sweetness and phenoliccomposition.

HA+B-RW and B-W were clustered together in the lower right quadrant, well separated from HA-RW (Figure 5A). This suggests that heating retentate with protease and then stabilizing with bentonite (0.28 g/L as wine volume) had less impact on wine chemical and sensory profiles than heating retentate with protease alone, compared with protein stabilization *via* traditional bentonite fining (at 1 g/L), i.e., B-W. In previous juice pasteurization trials, AGP flash pasteurization treatment of juice (at 75°C for 1 min) did not yield significant sensory differences based on triangle tests (7). Heating retentate at a lower temperature for a longer period (i.e., at 62°C for 10 min, rather than 75°C for 1 min) may result in sensory differences which are not perceived with heating juice due to the absence of fermentation-derived aromas in juice.

In general, compounds on the right of the PLSR plot contributed to the fruity aromas, herbaceous flavor, and sweet mouthfeel, and thus the overall flavor intensity of wines (Figure 5B). Two of the varietal thiols, 3-MH, and 3-MHA, were found to be closely correlated with one another (*R* = 0.957), but not with overall flavor intensity. Some (but not all) volatile acids, alcohols, and esters imparted desirable fruity and floral aromas (21) and these volatiles were abundant in HA+B-RW (Table 2 and Figure 5), which likely accounts for its high overall flavor intensity rating (Figure 5). Among the varietal thiols, only 4-MMP was significantly lower in HA+B-RW; their low detection threshold concentrations, together with the relatively high quantities of other volatiles (e.g., ethyl esters), resulted in tropical aroma and flavor ratings that were not significantly different from control wines based on RATA results. In comparison, H+B-RW and HA-RW had lower concentrations of volatile esters and alcohols (especially H+B-RW), which may explain their lower flavor intensity scores (Table 2 and Figure 5). Furthermore, the alcohol warmth perceived in HA-RW may also be attributable to the diminished overall flavor intensity and/or volatile diversity of this wine.

B-RW was positioned away from the other wines, at the top of the PLSR score plot, reflecting enhanced green apple aroma and herbaceous flavor (Figures 4, 5) and the lowest TA and alcohol levels of treated wines (Table 1), potentially due to the inevitable dilution associated with bentonite addition. The higher bentonite dose required to stabilize B-RW may have diminished some of the positive flavor attributes, as previously noted (5), thereby enabling the green apple character to become more distinct. This suggests that some aroma volatiles were also concentrated in retentate, possibly *via* complexation with macromolecules. The position of HA-RW and H+B-RW wines to the far left of the PLSR score plot (Figure 5A) was consistent with the expert panel’s tasting notes (which referenced riper fruit characters, a slight “cooked fruit” attribute and a lack of floral notes, relative to other wines).

The overall flavor intensity and alcohol heat had less impact on the separation of wines within the PLSR plot. HA-RW and RW were both perceived to have more alcohol heat character than the bentonite-treated wines as well as increased concentrations of flavonoids and total phenolics (Figures 5A,B). The higher total phenolics might influence the perception of alcohol heat, as seen in a previous study using model wine (32). Benzaldehyde and 3-methylbutanal are typically associated with oxidative character (20) and were also located in close proximity to alcohol heat (Figure 5B), so may also have influenced the perception of warmth. The correlation of benzaldehyde and 3-methylbutanal with alcohol heat was more apparent in Factor 3 (explaining 7% X variation and 29% Y variation), suggesting an underlying relationship.

PLSR confirmed that differences in both the basic wine chemistry and volatile composition of control and treated wines contributed to the perceived variation in wine sensory profiles. However, the sensory profiles of treated wines were alike (only three attributes showed statistically significant differences), as such, there were no perceived differences in terms of wine quality. The protein-stable wine derived from combined UF/heat/protease/bentonite treatment most closely resembled the wine stabilized *via* traditional bentonite fining in this study.

## Conclusion

Heat and protease treatment of the retentate generated from UF achieved significant removal of haze-forming proteins, with heat and protease decreasing protein concentrations by 54%. This fractionation and treatment enabled protein stabilization using less bentonite compared with traditional fining (0.28 and 1.0 g/L, respectively). Chemical and sensory analyses demonstrated that the combined UF/heat/protease treatment improved the heat stability of wine without significantly affecting wine composition or quality, relative to wine that was protein stabilized *via* traditional bentonite fining. The combined treatment retained flavor without introducing oxidative characters (perceivable browning or undesirable aromas/flavors), such that wine sensory profiles and quality ratings of UF-treated heat stable wines were comparable with bentonite-fined wine. These results confirm the combined treatment may offer a novel approach to protein stabilization which reduces bentonite use, without deleterious effects. Ideally, however, the use of bentonite would be eliminated altogether. UF of wine on a commercial scale can achieve a higher degree of permeation (i.e., > 90%), which enables further concentration of haze-forming proteins in a smaller volume of retentate. Optimization of heat/protease treatment at commercial scale is therefore the subject of further research. Techno-economic analysis is also required to establish the fiscal viability of this approach compared with traditional bentonite fining.

## Data Availability Statement

The original contributions presented in this study are included in the article/Supplementary Material, further inquiries can be directed to the corresponding author/s.

## Ethics Statement

The studies involving human participants were reviewed and approved by the University of Adelaide’s Human Research Ethics Committee (Approval No. H-2019-073). The participants provided their written informed consent to participate in this study.

## Author Contributions

YS: conceptualization, investigation, data curation, formal analysis, and writing—original draft, review and editing. DW: conceptualization, data curation, methodology, resources, and writing—review and editing. JM and RM: conceptualization, methodology, writing—review and editing, and supervision. DC: formal analysis and writing—review and editing. KW: conceptualization, methodology, writing—review and editing, resources, funding acquisition, and supervision. All authors contributed to the article and approved the submitted version.

## Conflict of Interest

DW declares a potential competing interest as the developer of VAF Memstar’s ultrafiltration system. The remaining authors declare that the research was conducted in the absence of any commercial or financial relationships that could be construed as a potential conflict of interest.

## Publisher’s Note

All claims expressed in this article are solely those of the authors and do not necessarily represent those of their affiliated organizations, or those of the publisher, the editors and the reviewers. Any product that may be evaluated in this article, or claim that may be made by its manufacturer, is not guaranteed or endorsed by the publisher.

## References

[1] Ratnayake S Stockdale V Grafton S Munro P Robinson A Pearson W Carrageenans as heat stabilizers of white wine. *Aust J Grape Wine Res.* (2019) 25:439–50. 10.1111/ajgw.12411

[2] Waters EJ Alexander G Muhlack R Pocock KF Colby C O’Neill BK Preventing protein haze in bottled white wine. *Aust J Grape Wine Res.* (2005) 11:215–25. 10.1111/j.1755-0238.2005.tb00289.x

[3] van Sluyter SC McRae JM Falconer RJ Smith PA Bacic A Waters EJ Wine protein haze: mechanisms of formation and advances in prevention. *J Agric Food Chem.* (2015) 63:4020–30. 10.1021/acs.jafc.5b00047 25847216

[4] Lira E Rodríguez-Bencomo JJ Salazar FN Orriols I Fornos D López F. Impact of bentonite additions during vinification on protein stability and volatile compounds of albariño wines. *J Agric Food Chem.* (2015) 63:3004–11. 10.1021/acs.jafc.5b00993 25751284

[5] Vincenzi S Panighel A Gazzola D Flamini R Curioni A. Study of combined effect of proteins and bentonite fining on the wine aroma loss. *J Agric Food Chem.* (2015) 63:2314–20. 10.1021/jf505657h 25665100

[6] Salazar FN Marangon M Labbé M Lira E Rodríguez-Bencomo JJ López F. Comparative study of sodium bentonite and sodium-activated bentonite fining during white wine fermentation: its effect on protein content, protein stability, lees volume, and volatile compounds. *Eur Food Res Technol.* (2017) 243:2043–54. 10.1007/s00217-017-2917-z

[7] Marangon M Van Sluyter SC Robinson EM Muhlack RA Holt HE Haynes PA Degradation of white wine haze proteins by aspergillopepsin I and II during juice flash pasteurization. *Food Chem.* (2012) 135:1157–65. 10.1016/j.foodchem.2012.05.042 22953838

[8] Mierczynska-Vasilev A Qi G Smith P Bindon K Vasilev K. Regeneration of magnetic nanoparticles used in the removal of pathogenesis-related proteins from white wines. *Foods.* (2020) 9:1. 10.3390/foods9010001 31861250 PMC7022247

[9] Sui Y McRae J Wollan D Muhlack R Godden P Wilkinson K. Use of ultrafiltration and proteolytic enzymes as alternative approaches for protein stabilization of white wine. *Aust J Grape Wine Res.* (2021) 27:234–45. 10.1111/ajgw.12475

[10] Comuzzo P Voce S Fabris J Cavallaro A Zanella G Karpusas M Effect of the combined application of heat treatment and proteases on protein stability and volatile composition of Greek white wines. *Oeno One.* (2020) 54:175–88. 10.20870/oeno-one.2020.54.1.2952

[11] Scrimgeour N Nordestgaard S Lloyd N Wilkes E. Exploring the effect of elevated storage temperature on wine composition. *Aust J Grape Wine Res.* (2015) 21:713–22. 10.1111/ajgw.12196

[12] Robinson AL Mueller M Heymann H Ebeler SE Boss PK Solomon PS Effect of simulated shipping conditions on sensory attributes and volatile composition of commercial white and red wines. *Am J Enol Vitic.* (2010) 61:337–47.

[13] Hopfer H Ebeler SE Heymann H. The combined effects of storage temperature and packaging type on the sensory and chemical properties of chardonnay. *J Agric Food Chem.* (2012) 60:10743–54. 10.1021/jf302910f 23035911

[14] Cejudo-Bastante MJ Hermosín-Gutiérrez I Pérez-Coello MS. Accelerated aging against conventional storage: effects on the volatile composition of chardonnay white wines. *J Food Sci.* (2013) 78:C507–13. 10.1111/1750-3841.12077 23488723

[15] Malletroit V Guinard JX Kunkee RE Lewis MJ. Effect of pasteurization on microbiological and sensory quality of white grape juice and wine. *J Food Process Preserv.* (1991) 15:19–29. 10.1111/j.1745-4549.1991.tb00151.x

[16] Iland PG Bruer N Edwards G Caloghiris S Willkes E. *Chemical Analysi of Grapes and Wine: Techniques and Concepts.* Campbelltown, SA: Patrick Iland Wine Promotions (2013).

[17] McRae JM Barricklow V Pocock KF Smith PA. Predicting protein haze formation in white wines. *Aust J Grape Wine Res.* (2018) 24:504–11. 10.1111/ajgw.12354

[18] Culbert JA McRae JM Condeì BC Schmidtke LM Nicholson EL Smith PA Influence of production method on the chemical composition, foaming properties, and quality of Australian carbonated and sparkling white wines. *J Agric Food Chem.* (2017) 65:1378–86. 10.1021/acs.jafc.6b05678 28128557

[19] Capone DL Ristic R Pardon KH Jeffery DW. Simple quantitative determination of potent thiols at ultratrace levels in wine by derivatization and high-performance liquid chromatography–tandem mass spectrometry (HPLC-MS/MS) analysis. *Anal Chem.* (2015) 87:1226–31. 10.1021/ac503883s 25562625

[20] Mayr CM Capone DL Pardon KH Black CA Pomeroy D Francis IL. Quantitative analysis by GC-MS/MS of 18 aroma compounds related to oxidative off-flavor in wines. *J Agric Food Chem.* (2015) 63:3394–401. 10.1021/jf505803u 25819472

[21] Wang J Capone DL Wilkinson KL Jeffery DW. Chemical and sensory profiles of rosé wines from Australia. *Food Chem.* (2016) 196:682–93. 10.1016/j.foodchem.2015.09.111 26593542

[22] Parr WV White KG Heatherbell DA. Exploring the nature of wine expertise: what underlies wine experts’ olfactory recognition memory advantage? *Food Qual Prefer.* (2004) 15:411–20. 10.1016/j.foodqual.2003.07.002

[23] Gawel R Godden PW. Evaluation of the consistency of wine quality assessments from expert wine tasters. *Aust J Grape Wine Res.* (2008) 14:1–8. 10.1111/j.1755-0238.2008.00001.x

[24] Danner L Crump AM Croker A Gambetta JM Johnson TE Bastian SE. Comparison of rate-all-that-apply and descriptive analysis for the sensory profiling of wine. *Am J Enol Vitic.* (2018) 69:12–21. 10.5344/ajev.2017.17052

[25] Di Gaspero M Ruzza P Hussain R Vincenzi S Biondi B Gazzola D Spectroscopy reveals that ethyl esters interact with proteins in wine. *Food Chem.* (2017) 217:373–8. 10.1016/j.foodchem.2016.08.133 27664648

[26] Sauvage FX Bach B Moutounet M Vernhet A. Proteins in white wines: thermo-sensitivity and differential adsorbtion by bentonite. *Food Chem.* (2010) 118:26–34. 10.1016/j.foodchem.2009.02.080

[27] Vernhet A Meistermann E Cottereau P Charrier F Chemardin P Poncet-Legrand C. Wine thermosensitive proteins adsorb first and better on bentonite during fining: practical implications and proposition of alternative heat tests. *J Agric Food Chem.* (2020) 68:13450–8. 10.1021/acs.jafc.0c00094 32142274

[28] Dordoni R Colangelo D Giribaldi M Giuffrida MG De Faveri DM Lambri M. Effect of bentonite characteristics on wine proteins, polyphenols, and metals under conditions of different pH. *Am J Enol Vitic.* (2015) 66:518–30. 10.5344/ajev.2015.15009

[29] Macheix JJ Sapis JC Fleuriet A Lee CY. Phenolic compounds and polyphenoloxidase in relation to browning in grapes and wines. *Crit Rev Food Sci Nutr.* (1991) 30:441–86. 10.1080/10408399109527552 1910524

[30] Recamales ÁF Sayago A González-Miret ML Hernanz D. The effect of time and storage conditions on the phenolic composition and colour of white wine. *Food Res Int.* (2006) 39:220–9. 10.1016/j.foodres.2005.07.009

[31] Barril C Rutledge DN Scollary GR Clark AC. Ascorbic acid and white wine production: a review of beneficial versus detrimental impacts. *Aust J Grape Wine Res.* (2016) 22:169–81. 10.1111/ajgw.12207

[32] Gawel R Smith PA Cicerale S Keast R. The mouthfeel of white wine. *Crit Rev Food Sci Nutr.* (2018) 58:2939–56. 10.1080/10408398.2017.1346584 28678530

[33] Vilela-Moura A Schuller D Mendes-Faia A Silva RD Chaves SR Sousa MJ The impact of acetate metabolism on yeast fermentative performance and wine quality: reduction of volatile acidity of grape musts and wines. *Appl Microbiol Biotechnol.* (2011) 89:271–80. 10.1007/s00253-010-2898-3 20931186

[34] Nichols DJ Cheryan M. Production of soy isolates by ultrafiltration: process engineering characteristics of the hollow fiber system. *J Food Process Preserv.* (1981) 5:103–18. 10.1111/j.1745-4549.1981.tb00625.x

[35] Susanto H Feng Y Ulbricht M. Fouling behavior during ultrafiltration of aqueous solutions of polyphenolic compounds. *J Food Eng.* (2009) 91:333–40. 10.1016/j.jfoodeng.2008.09.011

[36] Ulbricht M Ansorge W Danielzik I König M Schuster O. Fouling in microfiltration of wine: the influence of the membrane polymer on adsorption of polyphenols and polysaccharides. *Sep Purif Technol.* (2009) 68:335–42. 10.1016/j.seppur.2009.06.004

[37] Siebert KJ Troukhanova NV Lynn PY. Nature of polyphenol- protein interactions. *J Agric Food Chem.* (1996) 44:80–5. 10.1021/jf9502459

[38] Galanakis CM Markouli E Gekas V. Recovery and fractionation of different phenolic classes from winery sludge using ultrafiltration. *Sep Purif Technol.* (2013) 107:245–51. 10.1016/j.seppur.2013.01.034

[39] Okuda T Fukui M Takayanagi T Yokotsuka K. Characterization of major stable proteins in chardonnay wine. *Food Sci Technol Res.* (2006) 12:131–6. 10.3136/fstr.12.131

[40] Falconer RJ Marangon M Van Sluyter SC Neilson KA Chan C Waters EJ. Thermal stability of thaumatin-like protein, Chitinase, and invertase isolated from Sauvignon Blanc and Semillon juice and their role in haze formation in wine. *J Agric Food Chem.* (2010) 58:975–80. 10.1021/jf902843b 20014848

[41] Dufrechou M Poncet-Legrand C Sauvage FX Vernhet A. Stability of white wine proteins: combined effect of pH, ionic strength, and temperature on their aggregation. *J Agric Food Chem.* (2012) 60:1308–19. 10.1021/jf204048j 22224874

[42] Esteruelas M Poinsaut P Sieczkowski N Manteau S Fort MF Canals JM Characterization of natural haze protein in sauvignon white wine. *Food Chem.* (2009) 113:28–35. 10.1016/j.foodchem.2008.07.031

[43] Marangon M Van Sluyter SC Waters EJ Menz RI. Structure of haze forming proteins in white wines: vitis vinifera thaumatin-like proteins. *PLoS One.* (2014) 9:e113757. 10.1371/journal.pone.0113757 25463627 PMC4252030

[44] Lambri M Dordoni R Silva A De Faveri DM. Comparing the impact of bentonite addition for both must clarification and wine fining on the chemical profile of wine from chambave muscat grapes. *Int J Food Sci Technol.* (2012) 47:1–12. 10.1111/j.1365-2621.2011.02800.x

[45] Vincenzi S Crapisi A Curioni A. Foamability of prosecco wine: cooperative effects of high molecular weight glycocompounds and wine PR-proteins. *Food Hydrocoll.* (2014) 34:202–7. 10.1016/j.foodhyd.2012.09.016

[46] Marchal R Bouquelet S Maujean A. Purification and partial biochemical characterization of glycoproteins in a champenois chardonnay wine. *J Agric Food Chem.* (1996) 44:1716–22. 10.1021/jf9506592

[47] Dambrouck T Marchal R Cilindre C Parmentier M Jeandet P. Determination of the grape invertase content (using PTA- ELISA) following various fining treatments versus changes in the total protein content of wine. Relationships with wine foamability. *J Agric Food Chem.* (2005) 53:8782–9. 10.1021/jf051276z 16248585

[48] Cozzolino D Cynkar WU Shah N Smith PA. Can spectroscopy geographically classify Sauvignon Blanc wines from Australia and New Zealand? *Food Chem.* (2011) 126:673–8. 10.1016/j.foodchem.2010.11.005

[49] Swiegers JH Kievit RL Siebert T Lattey KA Bramley BR Francis IL The influence of yeast on the aroma of Sauvignon Blanc wine. *Food Microbiol.* (2009) 26:204–11. 10.1016/j.fm.2008.08.004 19171264

[50] Green JA Parr WV Breitmeyer J Valentin D Sherlock R. Sensory and chemical characterisation of Sauvignon Blanc wine: influence of source of origin. *Food Res Int.* (2011) 44:2788–97. 10.1016/j.foodres.2011.06.005

[51] Esteruelas M Kontoudakis N Gil M Fort MF Canals JM Zamora F. Phenolic compounds present in natural haze protein of Sauvignon white wine. *Int Food Res J.* (2011) 44:77–83. 10.1016/j.foodres.2010.11.010

[52] Marangon M Van Sluyter SC Neilson KA Chan C Haynes PA Waters EJ Roles of grape thaumatin-like protein and chitinase in white wine haze formation. *J Agric Food Chem.* (2011) 59:733–40. 10.1021/jf1038234 21189017

[53] de Bruijn J Loyola C Arumí JL Martínez J. Effect of non-protein factors on heat stability of Chilean Sauvignon Blanc wines. *Chilean J Agric Res.* (2014) 74:490–6. 10.4067/S0718-58392014000400017 27315006

[54] Dennis EG Keyzers RA Kalua CM Maffei SM Nicholson EL Boss PK. Grape contribution to wine aroma: production of hexyl acetate, Octyl acetate, and benzyl acetate during yeast fermentation is dependent upon precursors in the must. *J Agric Food Chem.* (2012) 60:2638–46. 10.1021/jf2042517 22332880

[55] Pedersen DS Capone DL Skouroumounis GK Pollnitz AP Sefton MA. Quantitative analysis of geraniol, nerol, linalool, and α-terpineol in wine. *Anal Bioanal Chem.* (2003) 375:517–22. 10.1007/s00216-002-1716-x 12610703

[56] Moyano L Zea L Moreno J Medina M. Analytical study of aromatic series in sherry wines subjected to biological aging. *J Agric Food Chem.* (2002) 50:7356–61. 10.1021/jf020645d 12452658

[57] Herbst-Johnstone M Nicolau L Kilmartin PA. Stability of varietal thiols in commercial Sauvignon Blanc wines. *Am J Enol Vitic.* (2011) 62:495–502. 10.5344/ajev.2011.11023

[58] Zhang X Kontoudakis N Clark AC. Rapid quantitation of 12 volatile aldehyde compounds in wine by LC-QQQ-MS: a combined measure of free and hydrogen-sulfite-bound forms. *J Agric Food Chem.* (2019) 67:3502–10. 10.1021/acs.jafc.8b07021 30811191

[59] Benkwitz F Tominaga T Kilmartin PA Lund C Wohlers M Nicolau L. Identifying the chemical composition related to the distinct aroma characteristics of New Zealand Sauvignon Blanc wines. *Am J Enol Vitic.* (2012) 63:62–72. 10.5344/ajev.2011.10074

[60] Gómez-Míguez MJ Cacho JF Ferreira V Vicario IM Heredia FJ. Volatile components of Zalema white wines. *Food Chem.* (2007) 100:1464–73. 10.1016/j.foodchem.2005.11.045

[61] Lin H Liu Y He Q Liu P Che Z Wang X Characterization of odor components of Pixian Douban (broad bean paste) by aroma extract dilute analysis and odor activity values. *Int J Food Prop.* (2019) 22:1223–34. 10.1080/10942912.2019.1636816

[62] Bueno M Marrufo-Curtido A Carrascón V Fernández-Zurbano P Escudero A Ferreira V. Formation and accumulation of acetaldehyde and Strecker aldehydes during red wine oxidation. *Front Chem.* (2018) 6:20. 10.3389/fchem.2018.00020 29492401 PMC5817066

