# Supplementary material for: Chemical and Sensory Profiles of Sauvignon Blanc Wine Following Protein Stabilization Using a Combined Ultrafiltration/Heat/Protease Treatment
Source: Front Nutr. 2022 Jun 29;9:799809. doi: 10.3389/fnut.2022.799809 (PMC9277391; doi:10.3389/fnut.2022.799809)
Supplement: Supplementary file 1 [file Data_Sheet_1.docx]

Supplementary Material


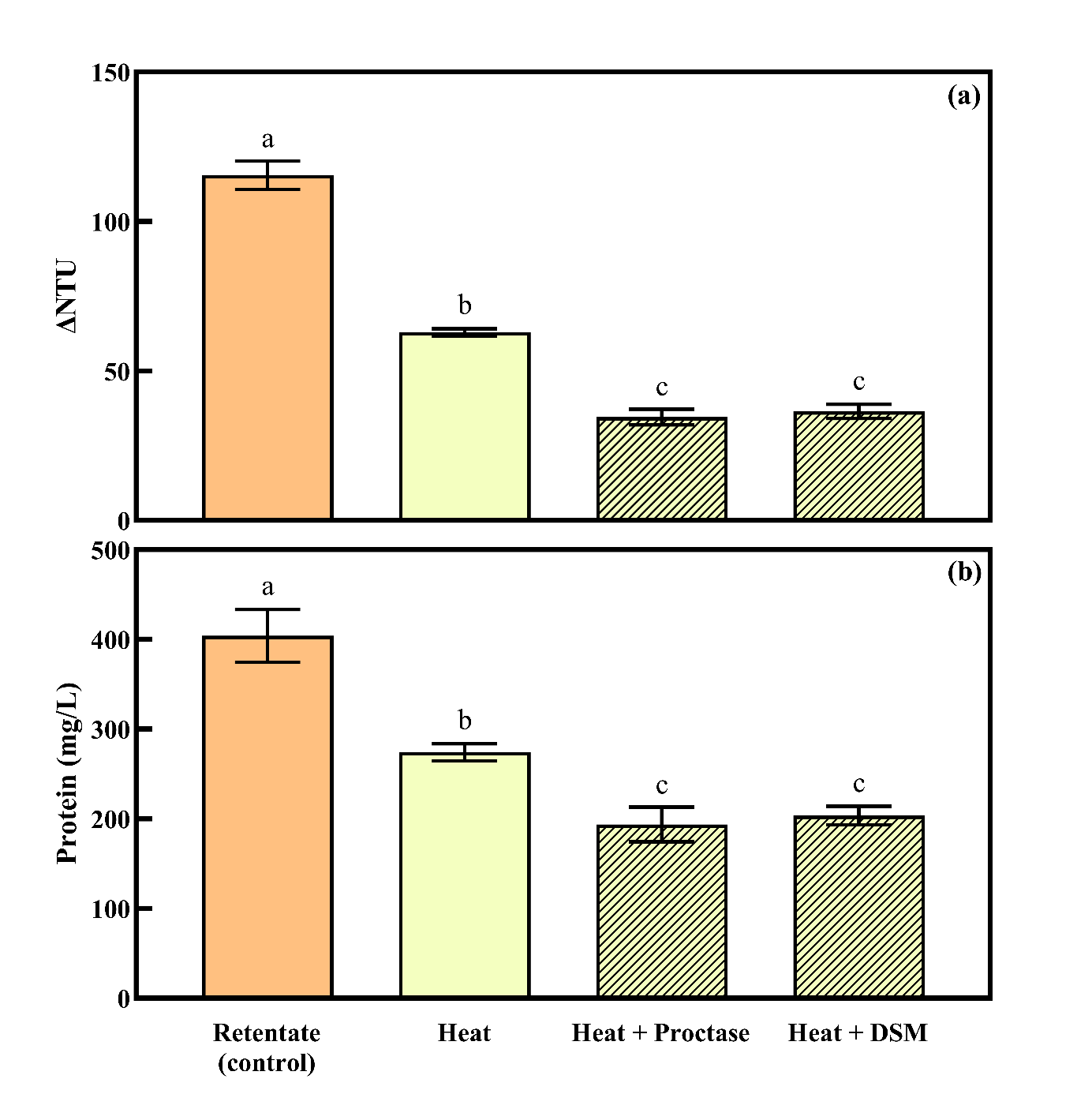
**\**

**Supplementary Figure 1.** Results from screening trial showing (a) heat stability (ΔNTU) and (b) haze-forming proteins in retentate before (control) and after heating with and without AGP enzymes (Proctase and DSM). Data are means of three replicates (± standard error). Different letters indicate statistically significant differences (one-way ANOVA, Tukey’s HSD, *P < 0.05*).


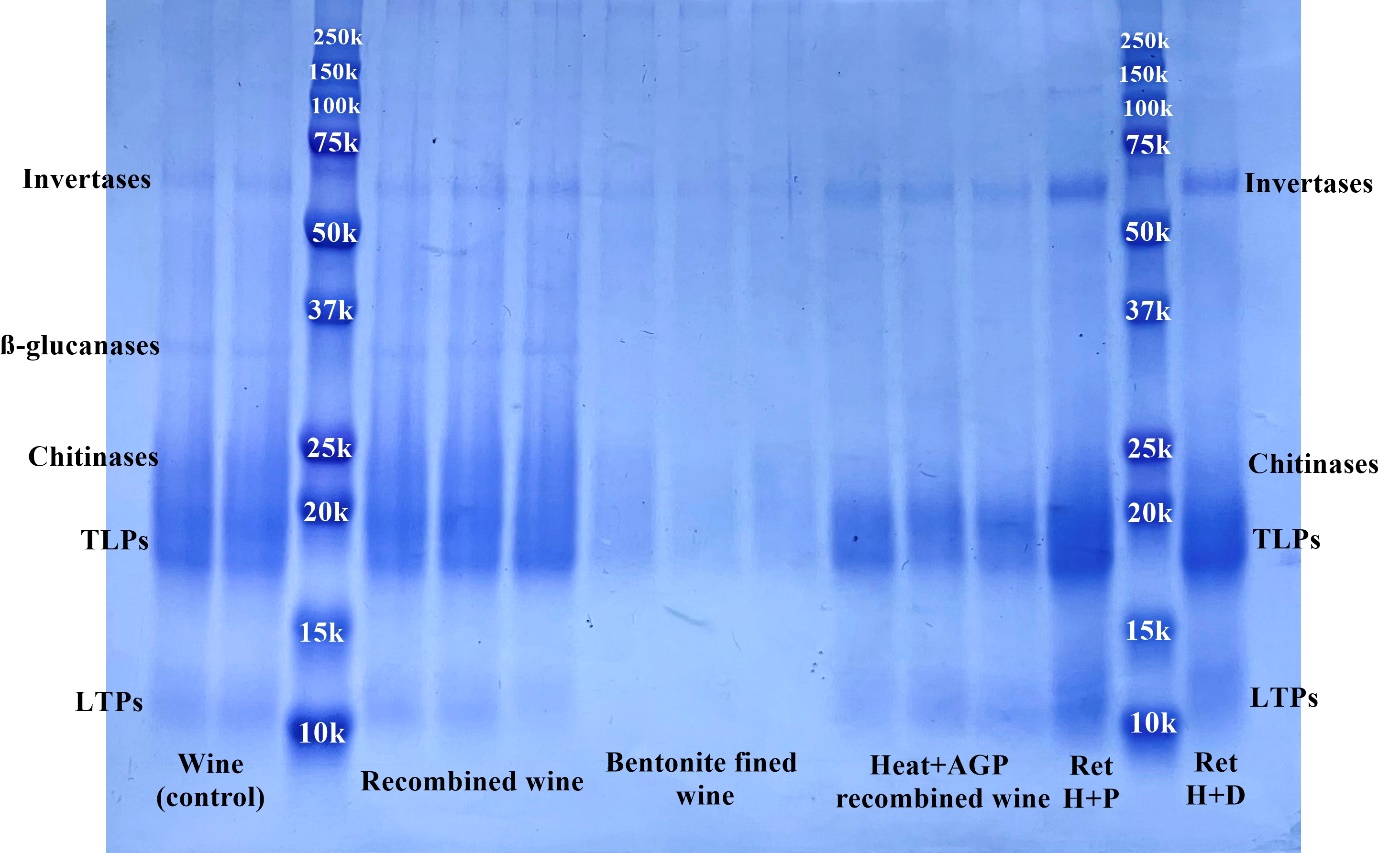


**Supplementary Figure 2.** SDS-PAGE result of wine proteins including lipid transfer proteins (LTPs), thaumatin like proteins (TLPs), chitinases, β-glucanases, and invertases in untreated wine (control) and treated wines: ultrafiltration recombined wine, bentonite fined wine and heat with DSM Aspergillopepsin (Heat + AGP) recombined wine. Molecular weight markers are noted in white.

**Supplementary Table 1.** Rate-All-That-Apply study results of attributes assessed on protein stabilization treated wines: bentonite fined (B-W) as a positive control, and recombined wines (RW) following ultrafiltration/heat/protease treatments; B denotes bentonite addition; H denotes heating (10 min at 62°C); A denotes Aspergillopepsin enzyme addition (DSM 0.05% v/v).

| **Attributes** | **B-W** | **RW** | **B-RW** | **H+B-RW** | **HA-RW** | **HA+B-RW** | ***P-value*** |
| --- | --- | --- | --- | --- | --- | --- | --- |
| ***Aroma*** |  |  |  |  |  |  |  |
| Overall intensity | 4.4 | 4.3 | 4.5 | 4.3 | 4.4 | 4.4 | *ns* |
| Citrus | 3.6 | 3.2 | 3.2 | 3.3 | 3.6 | 3.2 | *ns* |
| Tropical fruits | 3.6 | 3.3 | 3.9 | 3.5 | 3.8 | 3.6 | *ns* |
| Stone fruits | 2.9 | 2.7 | 3.1 | 3.1 | 3.2 | 2.6 | *ns* |
| Green apple | 2.1 b | 2.0 b | 2.8 a | 2.1 b | 1.9 b | 2.2 b | *0.012* |
| Floral | 2.5 | 2 | 2.3 | 2 | 2 | 2 | *ns* |
| Honey | 1.5 | 1.6 | 1.6 | 1.7 | 1.6 | 1.4 | *ns* |
| Herbaceous | 1.5 | 1.4 | 1.4 | 1.2 | 1.3 | 1.4 | *ns* |
| Grassy | 1.3 | 1.1 | 1.4 | 1.1 | 1.2 | 1.2 | *ns* |
| Leafy | 1.1 | 1 | 1.2 | 1 | 0.8 | 0.9 | *ns* |
| Green capsicum | 1 | 0.7 | 0.8 | 0.9 | 0.7 | 0.8 | *ns* |
| Cooked vegetable | 0.5 | 0.8 | 0.5 | 0.6 | 0.6 | 0.5 | *ns* |
| Banana | 1.4 a | 0.9 bc | 1.3 ab | 0.8 c | 0.9 abc | 1.0 abc | *0.107* |
| Boxwood/cat urine | 0.9 | 0.9 | 0.8 | 0.9 | 0.9 | 0.7 | *ns* |
| Solvent | 1.1 | 1 | 0.8 | 0.7 | 1.1 | 0.9 | *ns* |
| Toffee | 0.5 | 0.7 | 0.7 | 0.7 | 0.8 | 0.6 | *ns* |
| ***Flavor*** |  |  |  |  |  |  |  |
| Intensity | 4.2 abc | 4.1 bc | 4.3 ab | 3.9 c | 4.3 ab | 4.4 a | *0.009* |
| Citrus | 4.1 | 3.8 | 4.1 | 3.8 | 3.9 | 4 | *ns* |
| Tropical fruits | 3.2 | 3 | 3.2 | 3.2 | 3.3 | 3.5 | *ns* |
| Stone fruits | 2.6 | 2.6 | 2.6 | 2.8 | 2.7 | 2.8 | *ns* |
| Green apple | 2.4 | 2.6 | 2.6 | 2.4 | 2.2 | 2.5 | *ns* |
| Floral | 1.7 | 1.5 | 1.7 | 1.6 | 1.6 | 1.4 | *ns* |
| Honey | 1.3 | 1.2 | 1 | 1.4 | 1.1 | 1.1 | *ns* |
| Herbaceous | 1.5 ab | 1.2 b | 1.8 a | 1.1 b | 1.3 b | 1.4 ab | *0.101* |
| Grassy | 1.4 | 1.2 | 1.5 | 1.1 | 1.4 | 1.1 | *ns* |
| Leafy | 1 | 0.8 | 0.8 | 0.8 | 0.9 | 0.8 | *ns* |
| Green capsicum | 0.8 | 0.7 | 0.9 | 1 | 0.6 | 0.7 | *ns* |
| Cooked vegetable | 0.5 | 0.4 | 0.4 | 0.6 | 0.8 | 0.4 | *ns* |
| Banana | 0.7 | 0.6 | 0.8 | 0.5 | 0.7 | 0.6 | *ns* |
| Boxwood/cat urine | 0.8 | 0.7 | 0.9 | 0.7 | 0.6 | 0.7 | *ns* |
| Solvent | 0.8 | 0.7 | 1 | 0.6 | 1 | 0.7 | *ns* |
| Toffee | 0.5 | 0.6 | 0.7 | 0.3 | 0.5 | 0.6 | *ns* |
| ***Mouthfeel*** |  |  |  |  |  |  |  |
| Acidity | 4 | 4.2 | 4.1 | 4.1 | 4.1 | 4.2 | *ns* |
| Bitterness | 3 | 2.9 | 2.7 | 2.8 | 2.9 | 3 | *ns* |
| Sweetness | 2.9 a | 2.6 b | 2.9 ab | 2.7 b | 2.7 ab | 2.9 ab | *0.111* |
| Drying finish | 3 | 2.8 | 3.1 | 2.9 | 2.9 | 3 | *ns* |
| Astringency | 2.5 | 2.6 | 2.7 | 2.5 | 2.8 | 2.4 | *ns* |
| Alcohol heat/warmth | 3.2 b | 3.1 b | 3.2 b | 3.1 b | 3.5 a | 3.1 b | *0.0049* |

Data are means of panel rating (n = 54). Means followed by different letters are statistically significant (one-way ANOVA, Tukey’s HSD, *P < 0.05*); ns = not significant.

**Supplementary Table 2.** CIELab results of untreated wine (Control), wine treated via bentonite fining (B-W) as positive control, and recombined wines (RW) following ultrafiltration/heat/protease treatments; B denotes bentonite addition; H denotes heating (10 min at 62°C); A denotes Aspergillopepsin enzyme addition (DSM 0.05% v/v).

| **Sample** | **L** | **a** | **b** | **C** | **ΔE** |
| --- | --- | --- | --- | --- | --- |
| **Control** | 100.0 ± 0.1 | -0.09 ± 0.07 | 0.35 ± 0.19 | 0.38 ± 0.16 | - |
| **B-W** | 100.0 ± 0.1 | -0.03 ± 0.03 | 0.39 ± 0.15 | 0.39 ± 0.15 | 0.15 ± 0.02 |
| **RW** | 100.0 ± 0.1 | -0.02 ± 0.02 | 0.48 ± 0.01 | 0.48 ± 0.01 | 0.17 ± 0.02 |
| **B-RW** | 100.0 ± 0.0 | -0.03 ± 0.02 | 0.44 ± 0.01 | 0.45 ± 0.01 | 0.11 ± 0.01 |
| **H+B-RW** | 100.1 ± 0.0 | -0.03 ± 0.02 | 0.43 ± 0.02 | 0.43 ± 0.02 | 0.12 ± 0.01 |
| **HA-RW** | 100.1 ± 0.1 | -0.06 ± 0.03 | 0.40 ± 0.11 | 0.41 ± 0.10 | 0.13 ± 0.02 |
| **HA+B-RW** | 100.1 ± 0.0 | -0.02 ± 0.01 | 0.45 ± 0.02 | 0.45 ± 0.02 | 0.14 ± 0.03 |
| ***P-value*** | *ns* | *ns* | *ns* | *ns* | *-* |

Data are means of three replicates (± standard deviation). ns = not significant (one-way ANOVA, Tukey’s HSD, *P < 0.05*).

**Supplementary Table 3.** Qualitative information and method characteristics for volatile compounds determined by HS-SPME-GC-MS in control and treated wines.

| **Compound** | **CAS number** | **RT (min)** | **Ions (*m/z*)** | **R^2^** | **Internal standard** | **Aroma detection threshold (μg/L)** | **Odour quality** |
| --- | --- | --- | --- | --- | --- | --- | --- |
| Ethyl acetate | 141-78-6 | 4.79 | **61**, 88 | 0.9944 | d_4_-3-Methyl-1-butanol | 15000 | VA, nail polish, pineapple, varnish, balsamic (21) |
| Ethyl propanoate  (ethyl propionate) | 105-37-3 | 6.184 | 57, 75, **102** | 0.9988 | d_4_-3-Methyl-1-butanol | 21000 | Banana, apple (56) |
| Ethyl 2-methylpropanoate (ethyl isobutyrate) | 97-62-1 | 6.381 | 71, 88, **116** | 0.9925 | d_3_-Hexyl acetate | 56000 | Fruity (56) |
| Ethyl butanoate | 105-54-4 | 8.486 | 29, 71, **88** | 0.9957 | d_4_-3-Methyl-1-butanol | 20 | Strawberry, lactic/banana, strawberry (56) |
| 1-Propanol | 71-23-8 | 8.532 | **31**, 42, 59 | 0.9843 | d_13_-Hexanol | 500000 | Alcohol, pungent (21) |
| Ethyl 2-methylbutyrate  (ethyl 2-methylbutanoate) | 7452-79-1 | 9.024 | **57**, 85, 115 | 0.9906 | d_3_-Hexyl acetate | 1 | Fruity, anise, strawberry (21) |
| Ethyl 3-methylbutyrate (ethyl isovalerate) | 108-64-5 | 9.617 | 57, **88**, 130 | 0.9929 | d_3_-Hexyl acetate | 3 | Fruit (21) |
| Isobutanol  (2-methyl-1-propanol) | 78-83-1 | 11.057 | **31**, 55, 74 | 0.9928 | d_13_-Hexanol | 40000 | Wine, solvent, bitter (21) |
| 3-Methylbutyl acetate (isoamyl acetate) | 123-92-2 | 12.211 | 55, **70**, 87 | 0.9952 | d_4_-3-Methyl-1-butanol | 30 | Banana (21) |
| 1-Butanol | 71-36-3 | 13.195 | 31, 41, **56** | 0.9981 | d_4_-3-Methyl-1-butanol | 160000 | Medicinal (56) |
| 3-Methyl-1-butanol | 123-51-3 | 16.747 | 42, **55**, 70 | 0.9971 | d_4_-3-Methyl-1-butanol | 30000 | Harsh, nail polish, fusel (21) |
| Ethyl hexanoate  (ethyl caproate) | 123-66-0 | 18.039 | 60, **88**, 99, | 0.9939 | d_4_-3-Methyl-1-butanol | 14 | Apple peel, fruit (21) |
| Hexyl acetate | 142-92-7 | 20.213 | **43**, 56, 69 | 0.9982 | d_3_-Hexyl acetate | 670 | Fruit, floral (21) |
| Ethyl lactate | 97-64-3 | 24.341 | 29, **45**, 75 | 0.9959 | d_4_-3-Methyl-1-butanol | 146000 | Solvent (21) |
| 1-Hexanol | 111-27-3 | 24.987 | **56**, 69, 84 | 0.9984 | d_13_-Hexanol | 8000 | Green, grass, wood (21) |
| 3-Octanol | 589-98-0 | 27.587 | **59**, 83, 101 | 0.9977 | d_13_-Hexanol | N/A | Earthy, mushroom, herbal (56) |
| Ethyl octanoate  (ethyl caprylate) | 106-32-1 | 30.546 | **88,** 101, 127 | 0.9729 | d_13_-Hexanol | 20 | Melon, wood (21) |
| Acetic acid (use neat) | 64-19-7 | 30.749 | **43**, 45, 60 | 0.9921 | d_7_-Butyric acid | 200000 | Sour, vinegar (21) |
| 2-Ethyl-1-hexanol | 104-76-7 | 33.32 | **57**, 70, 98 | 0.9946 | d_13_-Hexanol | 8000 | Citrus, green (21) |
| Linalool | 78-70-6 | 36.885 | **71,** 121, 136 | 0.9994 | d_5_-Linalool | 15 | Flower, lavender (21) |
| 1-Octanol | 111-87-5 | 37.413 | **56**, 70, 84 | 0.9943 | d_13_-Hexanol | 0.7 | Chemical, metal, burnt (21) |
| Isobutanoic acid (isobutyric acid) | 79-31-2 | 37.724 | 43, **73**, 88 | 0.9786 | d_13_-Hexanol | 2300 ^c^ | N/A |
| Butanoic acid  (butyric acid) | 107-92-6 | 41.154 | 42, **60**, 73 | 0.9931 | d_13_-Hexanol | 2500 | Cheese, rancid (56) |
| Ethyl decanoate | 110-38-3 | 42.541 | **88,** 101, 155 | 0.9767 | d_4_-3-Methyl-1-butanol | 200 | Floral, soap (21) |
| 3-Methylbutanoic acid (Isovaleric acid) | 503-74-2 | 43.542 | 43, **60**, 87 | 0.9901 | d_13_-Hexanol | 1500 | Cheese, rancid (56) |
| Diethyl succinate | 123-25-1 | 44.414 | 101, **129,** 147 | 0.9939 | d_4_-3-Methyl-1-butanol | 1250000 | Wine, fruit (21) |
| α-Terpineol | 98-55-5 | 45.286 | **59**, 93, 136 | 0.9968 | d_13_-Hexanol | 250 | Oil, anise, spicy (21) |
| Ethyl 2-phenylacetate | 101-97-3 | 49.926 | 65, **91**, 164 | 0.9906 | d_13_-Hexanol | 650 | Fruit, sweet (21) |
| 2-Phenylethyl acetate | 103-45-3 | 51.441 | 65, 91, **104** | 0.9965 | d_5_-Phenylethanol | 250 | Jammy, plum, floral, fruity (21) |
| Hexanoic acid | 142-62-1 | 52.356 | **60**, 73, 87 | 0.999 | d_11_-Hexanoic acid | 420 | Leafy, wood, varnish (21) |
| Benzyl alcohol | 100-51-6 | 53.501 | 79, 91, **108** | 0.9995 | d_5_-Phenylethanol | 2000000 | Floral, rose, balsamic (60) |
| 2-Phenylethanol | 60-12-8 | 54.734 | 65, **91**, 122 | 0.9928 | d_5_-Phenylethanol | 14000 | Floral, rose (21) |
| Octanoic acid | 124-07-2 | 58.964 | **60**, 73, 101 | 0.9921 | d_15_-Octanoic acid | 500 | Butter, almond (21) |

Information of aroma detection threshold and descriptors are extracted from published work (21, 56, 60.

N/A = not available.

**Supplementary Table 4.** Concentrations (μg/L) of oxidative volatile compounds measured in control and treated wines, and their aroma detection thresholds, sensory descriptors and reported wine concentrations. Wine codes are untreated wine (Control), wine treated via bentonite fining (B-W) as a positive control, and recombined wines (RW) following ultrafiltration/heat/protease treatments; B denotes bentonite addition; H denotes heating (10 min at 62°C); A denotes Aspergillopepsin enzyme addition (DSM 0.05% v/v).

| Oxidative volatiles | Detection Threshold | Sensory descriptors | Reported concentration | Control | B-W | RW | B-RW | H+B-RW | HA-RW | HA+B-RW | P-value |
| --- | --- | --- | --- | --- | --- | --- | --- | --- | --- | --- | --- |
| 2-Methylbutanal | 4.4 (61) | Reagent-like, almond (61) | 4.9-45.3 (62) | 20.3 | 22.7 | 15.7 | 22.3 | 22.3 | 18.0 | 20.0 | ns |
| 3-Methylbutanal | 4.6 (20) | Malty, almond (20, 61) | 108-477 (58),  3.81-99.5 (20) | 19.0 abc | 22.0 abc | 13.7 c | 23.0 ab | 24.7 a | 15.0 bc | 20.3 abc | 0.0077 |
| Hexanal | 20 (20) | Green, grassy (20) | 3.7-18 (58), 0.7-2.1 (20) | 0.51 | 0.01 | 0.01 | 0.01 | 0.09 | 0.4 | 0.01 | ns |
| (*E*)-2-Hexenal | 4 (20) | Green apple (20) | 1.5 (58), 0.017-0.087 (20) | <0.01 | <0.01 | <0.01 | <0.01 | <0.01 | <0.01 | <0.01 | ns |
| Maltol | 5000 (20) | Caramel (20) | 79-165 (20) | <40 | <40 | <40 | <40 | <40 | <40 | <40 |  |
| (*E*)-2-Octenal | 3 (20) | Fatty, nutty (20) | 0.57 (58), 0.01-0.018 (20) | 0.2 | 0.2 | 0.01 | 0.01 | 0.12 | 0.01 | 0.13 | ns |
| 5-Methylfurfural | 2000 (20) | Sweet, bitter almond (20) | 33.4-46.6 (20) | <10 | <10 | <10 | <10 | <10 | <10 | <10 |  |
| (*E*)-2-Heptenal | 4.6 (20) | Soapy, fatty (20) | 2.3 (58), 0.1-0.112 (20) | 0.3 | 0.2 | 0.2 | 0.2 | 0.2 | 0.3 | 0.2 | ns |
| Methional | 0.5 (20) | Cooked potato-like (20) | 0.69-22.8 (20), 2.7-44 (58), 17.5-102.1 (62) | 0.7 | 0.7 | 0.5 | 0.6 | 0.6 | 0.6 | 0.5 | ns |
| Methionol | 1000 (20) | Sweet, potato-like (20) | 351-494 (20) | 405.3 a | 387.0 b | 382.0 bc | 370.0 c | 372.7 bc | 380.5 bc | 379.0 bc | <0.0001 |
| Benzaldehyde | 2000 (20) | Bitter, almond-like (20) | 2.6-45 (58) | 41.7 bc | 42.0 bc | 31.0 c | 67.7 ab | 94.0 a | 47.5 bc | 61.3 bc | 0.0002 |
| (*E*)-2-Nonenal | 0.17 (20) | Green, fatty, sawdust (20) | 0.27-0.94 (58), 0.01-0.054 (20) | 0.8 ab | 0.7 b | 0.7 b | 0.7 b | 0.7 b | 1.3 a | 0.8 ab | <0.0001 |
| 2-Phenylacetaldehyde | 1 (20) | Honey, floral (20) | 2.8-26.5 (20), 3.4-18 (58), 28.8-161.4 (62) | 18.0 a | 15.3 ab | 13.7 ab | 12.0 b | 13.3 ab | 13.5 ab | 12.0 b | 0.0388 |
| Furfural | 14100 (20) | Sweet, bread (20) | 1000-1311 (20),  25-1230 (58) | 165.7 a | 142.3 ab | 138.7 ab | 131.7 b | 144.3 ab | 163.5 ab | 152.3 ab | 0.0177 |
| Eugenol | 6 (20) | Clove, honey, wood (20) | 1.3-2.0 (20) | 4.6 a | 4.2 ab | 3.7 b | 3.7 b | 3.7 b | 3.8 b | 3.6 b | 0.0005 |

Data are means of three replicates (n = 3). Means followed by different letters are statistically significant (one-way ANOVA, Tukey’s HSD,
*P < 0.05*); ns = not significant. Information extracted from published work (20, 58, 61, 62).
